# Prenatal Stress Induces Changes in Behavior, HPA Axis, Inflammation, and Oxidative Stress in Adult Rats Offspring

**DOI:** 10.1007/s11064-026-04672-3

**Published:** 2026-02-28

**Authors:** Jorge M. Aguiar-Geraldo, Jefté Peper-Nascimento, José Henrique Cararo, Taise Possamai-Della, Alexandra I. Zugno, Anilkumar Pillai, João Quevedo, Samira S. Valvassori

**Affiliations:** 1 Translational Psychiatry Laboratory, Graduate Program in Health Sciences, University of Southern Santa Catarina (UNESC), Criciúma, SC Brazil; 2 Faillace Department of Psychiatry and Behavioral Sciences, McGovern Medical School, Center of Excellence on Mood Disorders, The University of Texas Health Science Center at Houston (UTHealth), Houston, TX USA; 3 Neuroscience Graduate Program, The University of Texas MD Anderson Cancer Center UTHealth Graduate School of Biomedical Sciences, Houston, TX USA; 4 Translational Psychiatry Program, Faillace Department of Psychiatry and Behavioral Sciences, McGovern Medical School, The University of Texas Health Science Center at Houston (UTHealth), Houston, TX USA; 5 Charlie Norwood VA Medical Center, Augusta, GA USA; 6 Center for Interventional Psychiatry, Faillace Department of Psychiatry and Behavioral Sciences, McGovern Medical School, The University of Texas Health Science Center at Houston (UTHealth Houston), Houston, TX USA

**Keywords:** Bipolar disorder, Schizophrenia, Inflammation, Prenatal exposure delayed effects, Hypothalamic-pituitary-adrenal axis

## Abstract

Prenatal stress is related to the development of psychiatric disorders involving inflammation, oxidative stress, and hypothalamic-pituitary-adrenal (HPA) axis. Therefore, the aim of the present study was to evaluate the effects of prenatal stress on behavior, inflammation, oxidative stress, and the HPA-axis in the dams and their offspring treated with lithium. Thirteen pregnant Wistar rats were exposed to a prenatal chronic unpredictable stress protocol from the 14th day of gestation until birth. At the 60th postnatal day (PND), a treatment protocol was carried out in the offspring with lithium (intraperitoneally – 47.5 mg/kg) or saline for seven days (twice a day). The behavior was assessed in the open field test to evaluate free movements. The dams (21 PND) and offspring (after open field) were euthanized, their brains were dissected in frontal cortex, hippocampus, and striatum, and the serum was collected. In the brain and/or serum, the levels of oxidative stress, inflammation, and HPA axis parameters were evaluated. Female offspring from stressed dams showed hyperactivity. Besides behavior alterations, offspring brain and serum showed an increase in pro-inflammatory cytokines, oxidative damage markers, and HPA axis hormones levels. Lithium administration only reduced the biochemical alterations. The prenatal stress protocol induced long-lasting behavior, inflammatory, oxidative stress, and HPA-axis alterations in the offspring which could underlie the development of psychiatric disorders.

**Supplementary Information:**

The online version contains supplementary material available at 10.1007/s11064-026-04672-3.

## Introduction

Maternal stress at pregnancy significantly influences the neurodevelopment of offspring in both animals and humans, with potential long-term implications for health and well-being [1–3]. The prenatal period represents a critical window for brain development, during which exposure to environmental stressors can induce enduring neurobiological changes in the offspring [4]. These alterations may increase the risk of neuropsychiatric disorders and hinder adaptive capacities later in life [5–7]. Conditions such as anxiety, depression, attention deficit hyperactivity disorder, and autism spectrum disorders have been linked to prenatal stress exposure [2, 3, 8, 9].

The “developmental programming hypothesis” provides an explanatory framework for these phenomena, postulating that fetal adaptation to maternal stress can reconfigure fundamental physiological and metabolic processes, with effects often persisting into adulthood [3]. In this context, the hypothalamic-pituitary-adrenal (HPA) axis and maternal cortisol levels emerge as central mediators of prenatal stress-induced changes [10, 11]. Maternal cortisol levels during pregnancy have been shown to predict offspring cortisol reactivity profiles in response to stressors [3, 12]. Elevated maternal glucocorticoid levels, triggered by stress, are associated with significant behavioral and neurotransmitter alterations in offspring [13], including disruptions in serotonergic and catecholaminergic pathways [14]. Therefore, evidence suggests that altered HPA axis functioning in offspring, caused by prenatal stress, may be one mechanism increasing their vulnerability to mental and behavioral health problems in adulthood [15–17].

One of the systems influenced by stress hormones during pregnancy is the immune system [9, 18]. The maternal systemic inflammatory state can induce an inflammatory response in the placenta, amniotic fluid, and fetus, leading to epigenetic modifications, microglial activation, neuroinflammation, and oxidative stress [19, 20]. These processes, often associated to mitochondrial dysfunction, can create a self-perpetuating damage cycle. Mediators of the maternal stress response, such as increased inflammation or cortisol production, impact redox balance in the embryonic environment [21]. Thus, these findings suggest that maternal stress may have long-lasting effects on the brain redox balance associated to inflammatory dysfunction [22].

These processes, encompassing HPA axis dysregulation, heightened inflammation, and oxidative stress, have been implicated in the pathophysiology of psychiatric disorders, including bipolar disorder. Such mechanisms contribute to altered neurotransmitter signaling, disruptions in neuroplasticity, and the persistence of neuroinflammatory states implicated in the pathophysiology of bipolar disorder [23–26]. The interplay between oxidative damage and chronic inflammation establishes a biochemical environment that perpetuates vulnerability to psychiatric conditions throughout life, particularly in individuals exposed to prenatal stress [25, 27]. However, further research is necessary to understand how prenatal stress contributes to the development of mood and neurodevelopmental disorders.

Preclinical animal models are essential for understanding the long-term effects of prenatal stress and their connection to poor offspring health [28]. In a previous study from our laboratory, we found that prenatal stress led to increased locomotor and exploratory activity in the open field test (face validity), hyperactivity of the HPA axis, and significant epigenetic changes in adult rat offspring (construct validity) [29]. Based on these findings, the present study investigates the potential of prenatal stress as a candidate model for studying bipolar disorder, with a particular focus on assessing lithium’s (Li) ability to mitigate these alterations (predictive validity).

We hypothesize that: (1) prenatal stress induces behavioral, inflammatory, and oxidative stress alterations in offspring, mediated by hypersensitization of the maternal HPA axis [2, 15, 30]; and (2) Li (a standard therapy for certain mood disorders) can reverse these molecular and behavioral dysfunctions due to its neuroprotective effects on plasticity, inflammation, and oxidative stress. Accordingly, this study aimed to evaluate the effects of prenatal stress on the maternal care, adult offspring behavior, HPA axis, inflammation, and oxidative stress in both dams and their adult offspring. Additionally, we examined the behavioral and neurochemical effects of the Li administration in adult offspring.

## Materials and Methods

### Animals

**Scheme 1:**
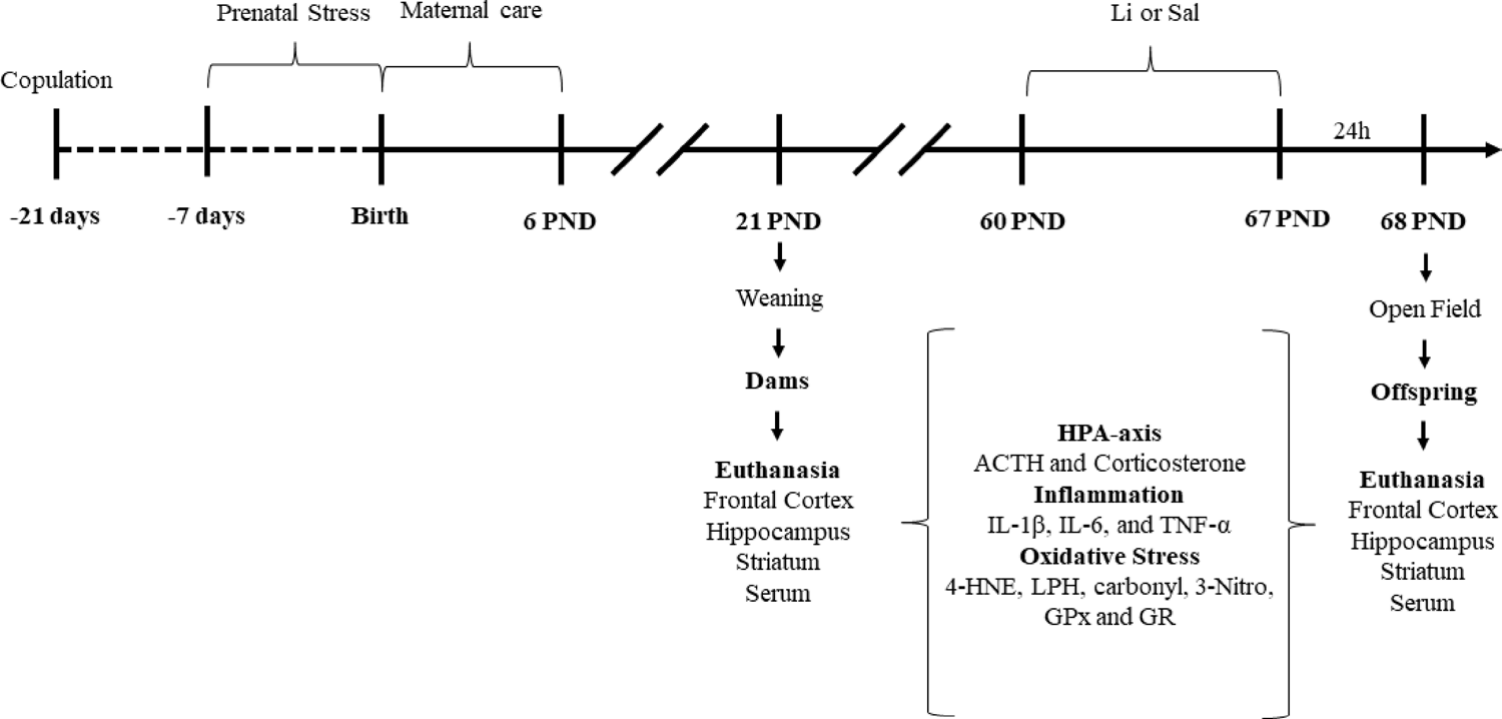
Experimental design for the present study (*PND* Postnatal days, *Li* Lithium, *HPA* Hypothalamic-pituitary-adrenal, *IL* Interleukin, *TNF* Tumor necrosis factor, *4-HNE* 4-hydroxynonenal, *3-Nitro* 3-nitrotyrosine, *LPH* Lipid peroxidation activity of lipid hydroperoxide, *GPx* Glutathione peroxidase activity, *GR* Glutathione reductase activity)

For the prenatal stress protocol, female Wistar rats (*Rattus norvegicus*, 60 days old) were obtained from the breeding colony at the University of Southern Santa Catarina (UNESC). Animals were kept in a 12-h light/dark cycle and received food and water *ad libitum*. This study followed the *Conselho Nacional de Controle de Experimentação Animal* (CONCEA) recommendations for animal care. In addition, all experiments were approved by the local ethics committee *Comissão de Ética no Uso de Animais* (CEUA) from UNESC under protocol number 014/2018. It is worthy to note that the animals were submitted to the acclimatization period to the laboratory conditions 30 min before each experiment. Experimental design is depicted in the Scheme 1.

### Prenatal Chronic Unpredictable Stress Protocol

Female rats were placed with male rats (one female and one male per box) for two days for mating. After the mating period, the male rats returned to the breeding colony. The females were housed in one per box (41 cm × 35 cm × 16 cm) until the start of the experiment.

On the 14th day of pregnancy, the dams were randomly assigned to control (*n* = 14) and stressed (*n* = 13) groups. The dams from the stressed group were submitted to the prenatal chronic unpredictable stress protocol, which consists of application of one stressor per day until de pups birth (21-22th day, adapted from Kinnunen et al. [31]), as follows: (1) Restraint in a polyvinyl chloride cylinder from 1 h (light cycle); (2) Restraint in a cold environment (4 °C) for 6 h (light cycle); (3) Forced swimming for 15 min (light cycle); (4) Overnight social stress induced by agglomeration (6 rats per box) (dark cycle); (5) Overnight food deprivation (dark cycle); (6) Light cycle for 24 h instead of the usual 12 h (light and dark cycle); (7) No stress, with could be regarded as one since the rats are “habituated” to receive a stressor each day; (8) Home box kept in a 45° angle (light cycle). This period was chosen because it is a crucial stage of neuronal development in rats, corresponding to the human second trimester of pregnancy [32]. The dams from the control group remained in their house box, and the only manipulation suffered by these rats was due to the daily colony’s maintenance protocol. There were in total 14 litters from the control group and 13 litters from the stressed group.

Immediately after the birth of the pups, the maternal care of control and stressed dams was evaluated for 6 days.

### Maternal Care Behavior Analysis

During 6 postnatal days (PND), the way dams care for their offspring (maternal care behavior) was evaluated. This protocol (developed by Myers and colleagues [33] modified by Popoola and colleagues [34] consists of five evaluations per day, three during the light period (06:00 am, 11:00 am, and 03:00 pm) and two during the dark period (06:00 pm and 10:00 pm). Each dam was evaluated for 5 s, with an interval of 3 min between the evaluations. In this way, each dam had its maternal care assessed 25 times per session (125 observations per day). The maternal care was evaluated through the following parameters: (1) Cleaning the offspring (licking and grooming); (2) Nursing; (3) Carrying the offspring to the nest; (4) In contact with the offspring without nursing; (5) Away from the offspring.

The different nursing postures were assigned to different categories: (a) passive nursing (when the rat is nursing the offspring lying sideways or on her back); (b) blanket nursing (when the mother is nursing above the offspring, without raising her back or paws); and (c) arched-back nursing (ABN) which was in turn divided into (1) ABN1: when the mother is just hovering over the offspring without arching the back; (2) ABN2: when the mother is hovering over the offspring with the back arched and front legs half-way extended (3) ABN3: when the mother is hovering over the offspring with the back arched and front legs extended.

At the end of the maternal care evaluation period, the dams and their offspring were kept without interventions until the 21 PND. At the 21 st PND, the offspring were separated from the dams and housed in five animals per box (41 cm × 35 cm × 16 cm, males and females separated), where they stayed without interventions until 60 PND.

### Pharmacological Intervention in the Offspring

At 60 PND, the offspring were randomly submitted to a Li (47.5 mg/kg – Li chloride diluted in saline) or saline (Sal – NaCl 0.9%) intraperitoneal (i.p.) administration for 7 days twice a day. The dosage of Li was based on a previous report [35]. This treatment protocol was chosen based on previous research from our group [36]. Therefore, the final experimental groups were: (1) Control + Sal; (2) Control + Li; (3) Prenatal stress + Sal; (4) Prenatal stress + Li. The final number of individuals *per* group was 10 for the behavior analysis and 5 for the biochemical analysis. This number was chosen based on a previous report from our research group [29]. On the day after the last injection, the behavior analysis of the offspring was carried out.

### Behavior Analysis of the Offspring

The offspring were submitted to the open field test to evaluate their locomotive, exploratory, stereotypic, and anxiety-like behavior. The behavior analysis was performed by a researcher blinded to experimental groups.

#### Open Field Test

The open field test was performed in a 40 cm × 60 cm × 50 cm plywood box with the frontal wall made of glass and the floor divided by black lines into 9 squares. Each animal is gently placed in the top left corner, facing the wall, and let explore the apparatus for 5 min. During this time, the number of crossing (locomotor behavior), rearing (exploratory behavior), visits to the center (risk behavior), fecal boli, and the time of grooming, sniffing (stereotyped behavior), and latency to leave the first square (anxiety-like behavior) are measured [37].

### Biochemistry Analysis

After the last behavior test, the dams and offspring were immediately submitted to euthanasia by decapitation without anesthesia, to not cause any alterations to the central nervous system. Rat brains were dissected to obtain the frontal cortex, hippocampus, and striatum. All brain structures were immediately kept frozen at − 80 °C for further analysis. From the cut made by decapitation, peripheral blood samples were collected in microtubes for subsequent analyses. The serum was obtained by centrifugation at 3000 *g* for 5 min and then frozen at − 80 °C until the beginning of the experiment. The parameters evaluated in the brain structures and serum were the levels of 4-hydroxynonenal (4-HNE), 3-nitrotyrosine (3-Nitro), carbonyl content, interleukin (IL)−1β, IL-6, tumor necrosis factor-alpha (TNF-α), and lipid peroxidation activity of lipid hydroperoxide (LPH), and the activity of glutathione peroxidase (GPx, EC 1.11.1.9) and glutathione reductase (GR, EC 1.8.1.7). In addition, the levels of ACTH and corticosterone were measured in the serum of the dams and offspring. All samples were standardized according to their protein concentrations using the Lowry et al. [38] method, adapted by Peterson [39].

#### Oxidative Stress Parameters

The lipid peroxidation parameters 4-HNE (µg/mL/protein - Cell Biolabs, Inc., San Diego, CA, USA; product code STA-838) and LPH (nmol/mg - Cayman Chemical Company, Ann Arbor, MI, USA; catalog no. 705003) were quantified by commercially available kits according to the manufacturer instructions. Carbonyl group content (nmol/mg/protein) was measured using a specific kit provided by Cell Biolabs (OxiSelect™ Protein Carbonyl ELISA Kit; catalog no. STA-310). 3-Nitro (µg/mg/protein) quantitation was performed using the assay kit by Cell Biolabs (San Diego, CA, USA; product code STA-305). After incubating the sample for a short period, the anti-3-nitrotyrosine antibody was added, followed by a secondary horseradish peroxidase (HRP)-conjugated antibody. 3-Nitrotyrosine levels in the samples were determined by comparison to a standard curve.

Activity of the enzymes GPx (Cayman Chemical, Paulinia, Brazil; item no.703102) and GR (Cayman Chemical, Paulinia, Brazil; item no.703202) were measured by the oxidation of nicotinamide adenine dinucleotide phosphate (NADPH), following the manufacturer instructions.

#### Inflammation Markers

The brain samples were homogenized in a phosphate-buffered saline extraction solution containing aprotinin (100 mg of tissue per 1 mL). Commercially available ELISA kits measured the concentrations of IL-1β, IL-6, and TNF-α, following the instructions supplied by the manufacturer (DuoSet; R&D Systems, Minneapolis, MN, US; product codes R6000B, RLB00, and RTA00). Data are shown in pg/100 mg tissue (for each brain structure) and as pg/mL serum.

#### Levels of Corticosterone and ACTH

Corticosterone levels were measured using enzyme immunoassay (EIA) kits (from Diagnostic Products Corporation, Los Angeles, CA, USA). ACTH serum concentrations were determined using commercially available radioimmunoassay kits (from Diagnostic Products Corporation) for animals.

### Statistical Analysis

All data were submitted to the Shapiro-Wilk test for normality. Outliers were excluded using the ROUT methodology. The number of exclusions was limited to a maximum of 1 per group. The outlier removal excluded animals from the parameters Open Field (Crossing, Rearing, Latency, and Center), for biochemical analysis (3-Nitro). Student’s *t*-test (for parametric data) and the Mann-Whitney test (for nonparametric data) were used to evaluate the maternal care and biochemistry data from the dams. For the offspring’s data, the three-way analysis of variance (ANOVA) followed by Tukey’s post hoc test was used for the parametric data. The Kruskal-Wallis test was used for nonparametric data, followed by Dunn’s post hoc. Data for the offspring (as parametric) were presented in bar graphs, where the bar represents mean ± standard deviation. As non-parametric, data were presented in a box-plot graph illustrating the median ± maximum (max) and minimum (min) values. All values from the biochemical analysis of the dams were presented in a table with mean ± standard deviation (for parametric) or median (CI) for nonparametric data (Table 1). *p* value ≤ 0.05 was considered significant. All statistical analyses were performed by GraphPad Prism software version 10.

**Table 1:** Data from the biochemistry analysis of the dams

	Mean ± SD; Median (CI)							
	Serum		Frontal Cortex		Hippocampus		Striatum	
	Control	Stress	Control	Stress	Control	Stress	Control	Stress
HPA axis								
ACTH	14.41 ± 2.68	12.81 ± 1.89	-	-	-	-	-	-
Corticosterone	12.89 ± 2.20	12.26 ± 2.90	-	-	-	-	-	-
Inflammation								
IL-1β	1781 ± 129.3	2196 ± 880.3	**2484 ± 424**,**8**	**1384 ± 290**,8*	2586 ± 495.5	2221 ± 434.2	2333 ± 456.8	2082 ± 417.3
IL-6	2809 ± 816.0	2035 ± 806.8	2416 ± 357.2	2439 ± 706.3	2274 ± 298.1	2131 ± 459.9	1913 ± 354.2	1834 ± 350.2
TNF-α	**5130 ± 1096**	8358 ± 888.6*	4567 ± 1140	4470 ± 1470	**2698 ± 568.9**	3344 ± 261.2*	4037 ± 524.2	3910 ± 551.9
Oxidative Stress								
4-HNE	**2.92 ± 0.52**	5.25 ± 0.69*	2.93 ± 0.30	2.83 ± 0.57	2.15 (2.05–2.94)	2.05 (1.98–2.36)	2.51 ± 0.36	3.04 ± 0.39
LPH	**2.54 ± 0.13**	5.72 ± 1.05*	3.13 ± 0.23	3.25 ± 0.82	3.05 ± 0.28	2.84 ± 0.91	3.08 ± 0.69	2.90 ± 0.47
Carbonyl	**43.24 ± 3.15**	67.75 ± 4.53*	42.08 ± 2.15	44.07 ± 7.86	48.42 ± 5.00	46.50 ± 7.01	37.21 ± 7.15	43.41 ± 6.82
3-Nitro	**0.26 ± 0.041**	0.48 ± 0.029*	**0.24 (0.23–0.36)**	0.16 (0.12–0.21)*	0.29 (0.23–0.34)	0.25 (0.23–0.36)	0.28 ± 0.036	0.30 ± 0.033
GPx	53.73 ± 7.97	45.18 ± 11.92	42.80 ± 7.05	44.68 ± 16.32	40.42 ± 10.52	52.12 ± 13.41	**46.85 ± 6.10**	31.86 ± 12.81*
GR	42.49 ± 15.47	38.85 ± 9.03	28.49 ± 7.42	39.14 ± 8.54	43.31 ± 7.48	34.35 ± 21.81	33.23 ± 7.32	44.95 ± 5.74*

Bold values indicate statistical difference between groups

Data from the analysis of the HPA axis, oxidative stress, and inflammation of the frontal cortex, hippocampus, striatum, and serum of control and stressed dams. Parametric data are presented as mean ± SD evaluated with the student’s t-test and nonparametric data are presented as median (CI) evaluated with the Mann-Whitney U test. HPA-axis are presented in pg/mL. Inflammation markers in the brain structures are presented in pg/100 mg of tissue and serum in pg/mL. Oxidative stress markers are presented in: 4-HNE = ng/mL/protein; LPH =; Carbonyl =; 3-Nitro =; GPx and GR = pmol/min/mL. * *p* ≤ 0.05 when compared to the control group

## Results

**Fig. 1:**
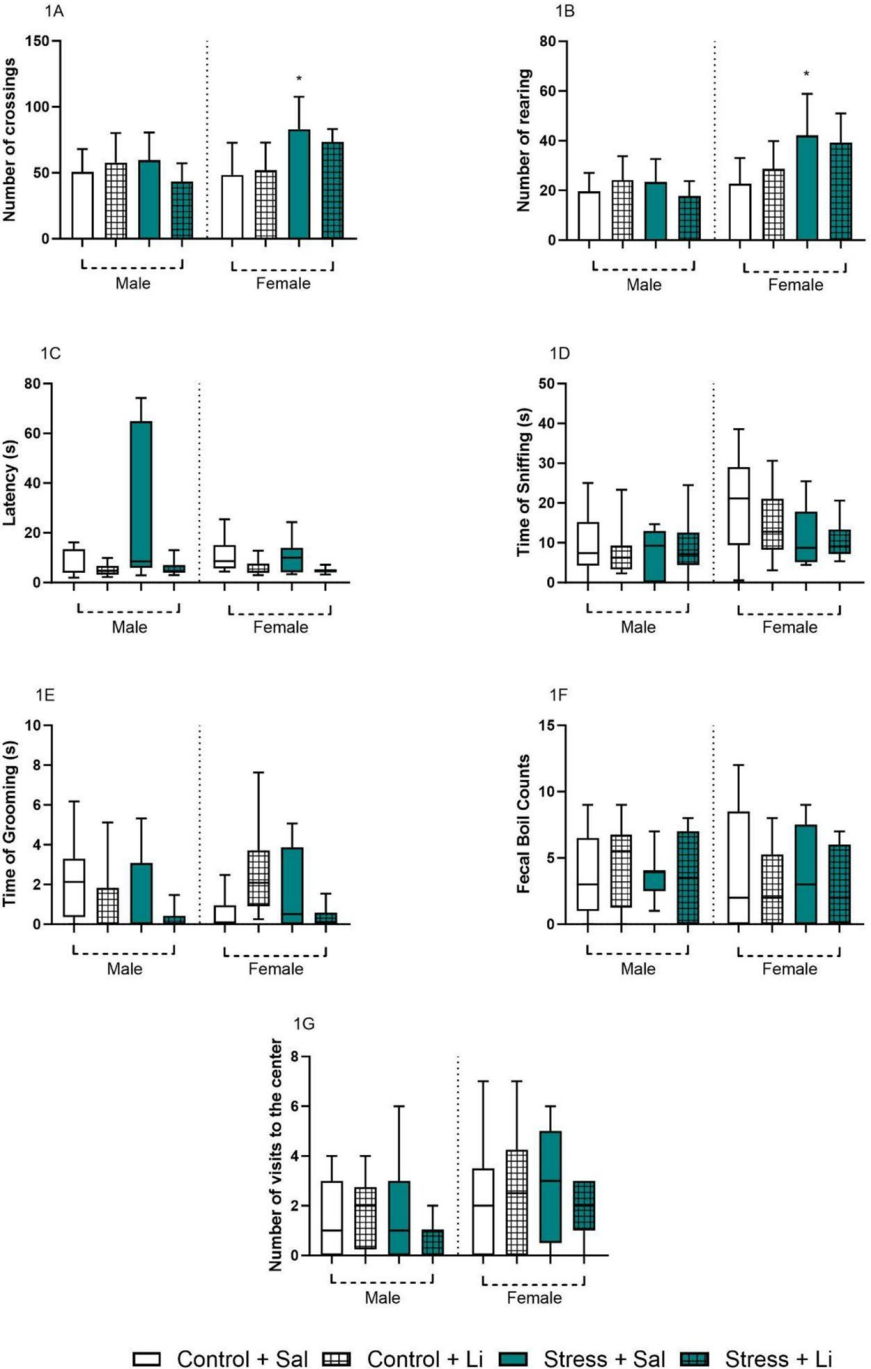
Data from the open field test of the offspring exposed to prenatal stress and treated with lithium (Li) in adulthood (60 days old). Data is presented as columns representing mean and standard deviation or box-plot representing median and min/max values according to distribution. Data presented as columns were evaluated using three-way ANOVA followed by Tukey’s post hoc test and data presented as box-plot were evaluated using the Kruskal-Wallis *H* test followed by Dunn’s post hoc test. *p < 0.05 as compared to Control + Sal group of the same sex

To assess the effects of Li on the behavior alterations, an open-field test was performed (Fig. 1). The graphs demonstrate that we successfully reproduced data from our previous study [29]. Regarding the crossing parameter, three-way ANOVA revealed a significant difference in the sex [F_(1,70)_ = 6.328, *p* = 0.0142], prenatal stress [F_(1,70)_ = 7.818, *p* = 0.0067], sex vs. prenatal stress [F_(1,70)_ = 11.53, *p* = 0.0011], and prenatal stress vs. treatment [F_(1,70)_ = 4.018, *p* = 0.0489]. No difference was found in the treatment [F_(1,70)_ = 0.6964, *p* = 0.4068], sex vs. treatment [F_(1,70)_ = 0.01995, *p* = 0.8881], and the interaction [F_(1,70)_ = 0.2848, *p* = 0.5953]. Tukey’s post hoc test indicated that females who were exposed to prenatal stress presented an increased number of crossings as compared to Control + Sal group. Additionally, Li could not fully revert these alterations found in the female offspring. No significant differences were found in the male offspring. Similarly, in the number of rearing parameter, three-way ANOVA demonstrated a significant difference regarding the sex of the offspring [F_(1,71)_ = 24.65, *p* < 0.0001], prenatal stress [F_(1,71)_ = 8.077, *p* = 0.0058], and sex vs. prenatal stress [F_(1,71)_ = 11.44, *p* = 0.0012]. No differences were found in treatment [F_(1,71)_ = 0.04801, *p* = 0.8272], sex vs. treatment [F_(1,71)_ = 0.1848, *p* = 0.6686], prenatal stress vs. treatment [F_(1,71)_ = 3.950, *p* = 0.0507], and the interaction [F_(1,71)_ = 0.01650, *p* = 0.8981]. According to Tukey’s post hoc, the female offspring of dams subjected to stress presented increased levels of rearings, compared to the Control + Sal group – an alteration not reversed by the Li administration. No significant differences were found regarding the male offspring.

In the open field test, the latency time to move from the first square was measured, and the Kruskal-Wallis *H* test revealed no significant differences between groups [*H* = 13.89, *p* = 0.0532]. Similar results were found regarding the time of sniffing [*H* = 12.37, *p* = 0.0891], fecal boil counts [*H* = 2.698, *p* = 0.9115], and visits to the center of the apparatus [*H* = 6.990, *p* = 0.4300]. Regarding the time of grooming, the Kruskal-Wallis test revealed a significant difference [*H* = 20.45, *p* = 0.0047], however, Dunn’s post hoc did not indicate any significant difference in comparison to the Control + Sal group in both sexes. Behavior analysis of maternal care is depicted in the Supplementary Fig. 1.

**Fig. 2:**
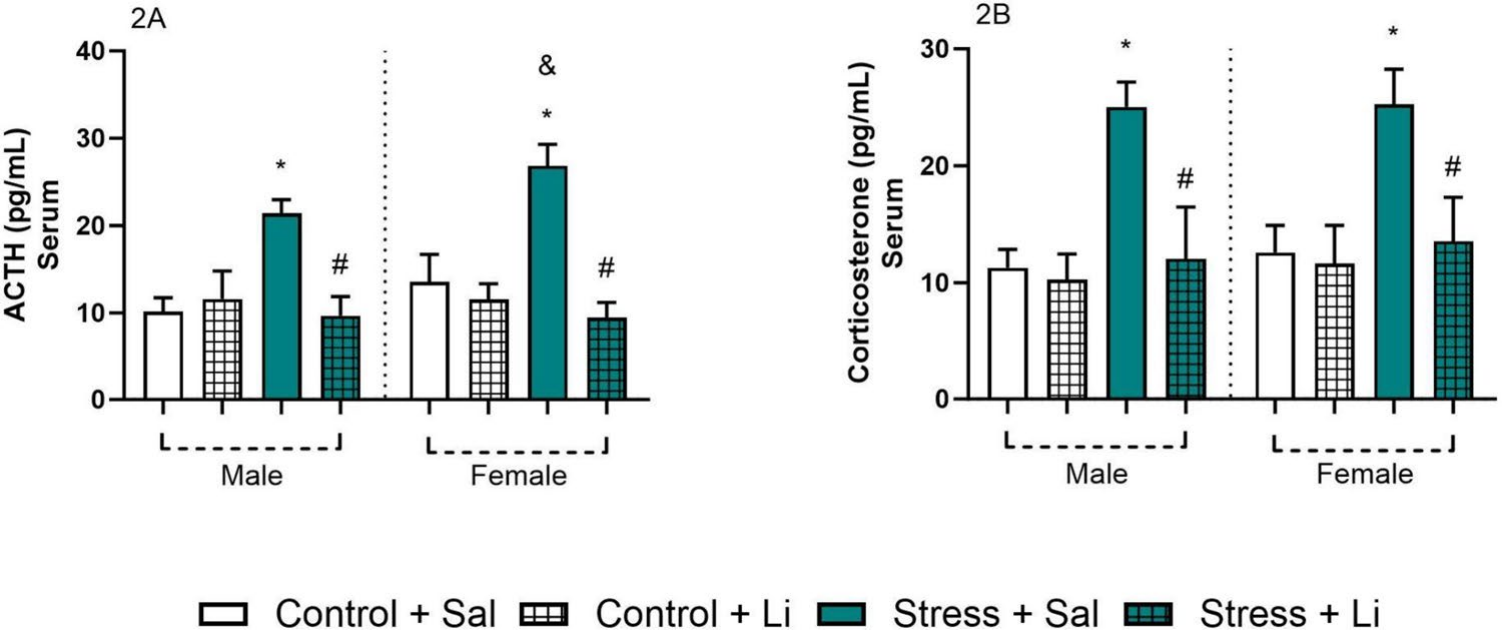
Serum levels of ACTH and corticosterone of the offspring from dams exposed to prenatal stress and treated with lithium (Li) at adulthood (60 days old). Data are presented as columns representing mean and standard deviation of mean. Three-way ANOVA followed by Tukey’s post hoc test. *p < 0.05 compared to Control + Sal of the same sex; #*p* < 0.05 compared to Stress + Sal of the same sex; &*p* < 0.05 compared with the same group of the opposite sex

With the goal of assessing the impact of prenatal stress and the effects of Li on the HPA axis of the offspring, the serum corticosterone and ACTH levels were evaluated (Fig. 2). Regarding the levels of ACTH, three-way ANOVA revealed a significant difference in the sex of the offspring [F_(1,32)_ = 8.75, *p* = 0.0058], prenatal stress [F_(1,32)_ = 49.77, *p* < 0.0001], treatment [F_(1,32)_ = 103.6, *p* < 0.0001], sex vs. treatment [F_(1,32)_ = 9.704, *p* = 0.0039], and prenatal stress vs. treatment [F_(1,32)_ = 96.77, *p* < 0.0001]. No significant differences were found in the sex vs. prenatal stress [F_(1,32)_ = 0.4409, *p* = 0.5115], and the interaction [F_(1,32)_ = 0.5696, *p* = 0.4559]. Tukey’s post hoc showed that, in both sexes, the levels of ACTH were increased in the offspring of stressed dams, and that Li reverted such alteration. Interestingly, the levels of ACTH were significantly higher in the female offspring exposed to prenatal stress, when compared to the male counterparts. Regarding the levels of corticosterone, three-way ANOVA indicated significant differences in prenatal stress [F_(1,32)_ = 64.31, *p* < 0.0001], treatment [F_(1,32)_ = 50.30, *p* < 0.0001], and prenatal stress vs. treatment [F_(1,32)_ = 36.60, *p* < 0.0001], However, no differences were found in the sex [F_(1,32)_ = 1.346, *p* = 0.2546], sex vs. prenatal stress [F_(1,32)_ = 0.06000, *p* = 0.8081], sex vs. treatment [F_(1,32)_ = 0.1220, *p* = 0.7291], and the interaction [F_(1,32)_ = 0.1014, *p* = 0.7522]. According to Tukey’s post hoc test, the offspring of both sexes from dams exposed to prenatal stress presented increased levels of corticosterone as compared to Control + Sal. This alteration was significantly reverted by the administration of Li at adulthood.

**Fig. 3:**
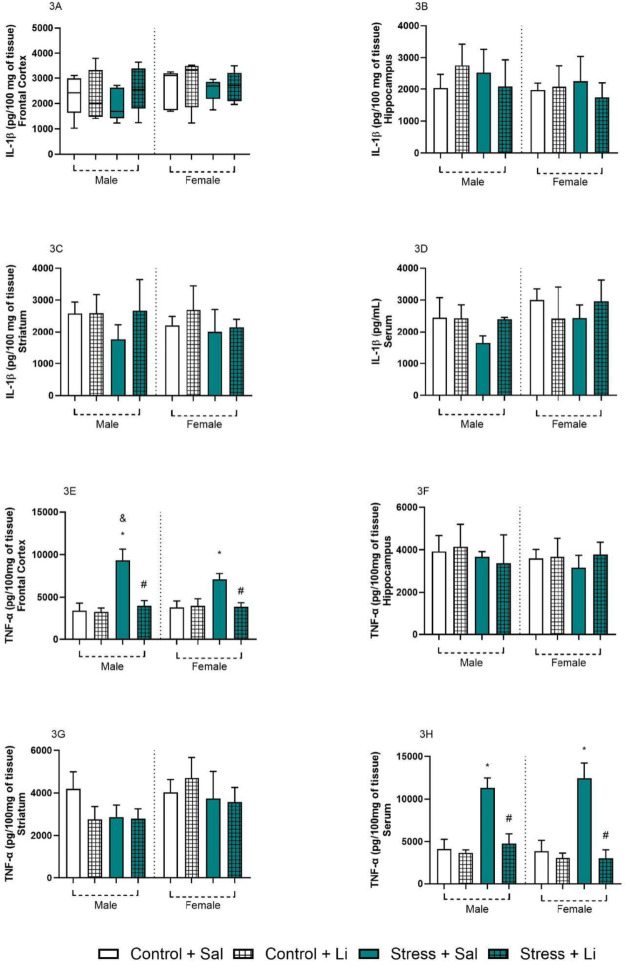
Brain and serum levels of interleukin (IL)−1β and tumor necrosis factor alpha (TNF)-α of the offspring from dams exposed to prenatal stress and treated with lithium (Li) at adulthood (60 days old). Data are presented as columns representing mean and standard deviation of mean. Three-way ANOVA followed by Tukey’s post hoc test. *p < 0.05 as compared to Control + Sal group of the same sex; #*p* < 0.05 as compared to Stress + Sal of the same sex; &*p* < 0.05 as compared to the same group of the opposite sex

To further evaluate the effects of prenatal stress in the offspring, and the effects of Li, the levels of inflammatory cytokines were evaluated in the brain and serum (Fig. 3). Regarding the levels of IL-1β in the frontal cortex, Kruskal-Wallis indicated no significant differences between groups [*H* = 4.809, *p* = 0.6833]. In the hippocampus, three-way ANOVA showed no significant differences regarding the sex of the offspring [F_(1,32)_ = 2.725, *p* = 0.1086], prenatal stress [F_(1,32)_ = 0.08263, *p* = 0.7756], treatment [F_(1,32)_ = 0.08263, *p* = 0.7756], sex vs. prenatal stress [F_(1,32)_ = 0.02404, *p* = 0.8778], sex vs. treatment [F_(1,32)_ = 0.7360, *p* = 0.3973], and the interaction [F_(1,32)_ = 0.7360, *p* = 0.3973]. Only prenatal stress vs. treatment showed a significant alteration [F_(1,32)_ = 4.741, *p* = 0.0369]. Similar effects were found in the striatal levels of IL-1β, where no differences were observed in the sex [F_(1,32)_ = 0.5212, *p* = 0.4756], prenatal stress [F_(1,32)_ = 3.930, *p* = 0.0561], treatment [F_(1,32)_ = 4.114, *p* = 0.0509], sex vs. prenatal stress [F_(1,32)_ = 2.518e-6, *p* = 0.9987], sex vs. treatment [F_(1,32)_ = 0.1676, *p* = 0.6850], prenatal stress vs. treatment [F_(1,32)_ = 0.5585, *p* = 0.4603], and the interaction [F_(1,32)_ = 2.640, *p* = 0.1140]. The serum levels of this cytokine were also not different between groups, and three-way ANOVA showed no significant difference in prenatal stress [F_(1,30)_ = 1.463, *p* = 0.2359], treatment [F_(1,30)_ = 0.8935, *p* = 0.3521], sex vs. prenatal stress [F_(1, 30)_ = 1.345, *p* = 0.2554] sex vs. treatment [F_(1,30)_ = 1.277, *p* = 0.2674], and the interaction [F_(1,30)_ = 0.2204, *p* = 0.6421]. Significant differences were found in the sex [F_(1,30)_ = 7.197, *p* = 0.0118], and prenatal stress vs. treatment [F_(1,30)_ = 6.982, *p* = 0.0130]. For all structures evaluated, the post hoc tests showed no significant differences between groups.

Regarding the levels of TNF-ɑ in the frontal cortex, three-way ANOVA demonstrated significant differences in prenatal stress [F_(1,32)_ = 89.93, *p* < 0.0001], treatment [F_(1,32)_ = 67.59, *p* < 0.0001], sex vs. prenatal stress [F_(1,32)_ = 10.85, *p* = 0.0024], sex vs. treatment [F_(1,32)_ = 5.32, *p* = 0.0277], and prenatal stress vs. treatment [F_(1,32)_ = 68.98, *p* < 0.0001]. No significant differences were found in sex [F_(1,32)_ = 1.400, *p* = 0.2454] and the interaction [F_(1,32)_ = 2.754, *p* = 0.1068]. Tukey’s post hoc indicated that, in both sexes, TNF-ɑ was increased in the offspring of stressed dams in the frontal cortex, and that the treatment with Li reverted all effects. Additionally, the levels of TNF-ɑ were higher in males compared to females in the Stress + Sal group. A similar effect was observed in the serum, where three-way ANOVA indicated a significant difference in prenatal stress [F_(1,32)_ = 138.1, *p* < 0.0001], treatment [F_(1,32)_ = 145.4, *p* < 0.0001], sex vs. treatment [F_(1,32)_ = 5.185, *p* = 0.0296], prenatal stress vs. treatment [F_(1,32)_ = 106.8, *p* < 0.0001]. No differences between groups were found regarding sex [F_(1,32)_ = 1.094, *p* = 0.3035], sex vs. prenatal stress [F_(1,32)_ = 0.03733, *p* = 0.8480], and the interaction [F_(1,32)_ = 3.082, *p* = 0.0887]. According to Tukey’s post hoc, TNF-ɑ was higher in the prenatal Stress + Sal groups as compared to control group, in both sexes. Also, Li reverted all these alterations. Lastly, no differences were observed in the hippocampus and striatum, according to ANOVA followed by Tukey’s post hoc [*Hippocampus*: sex F_(1,32)_ = 0.8115, *p* = 0.3744; prenatal stress F_(1,32)_ = 1.786, *p* = 0.1908; treatment F_(1,32)_ = 1.786, *p* = 0.1908; sex vs. prenatal stress F_(1,32)_ = 0.4751, *p* = 0.4956; sex vs. treatment F_(1,32)_ = 0.5733, *p* = 0.4545; prenatal stress vs. treatment F_(1,32)_ = 0.0004293, *p* = 0.9836; interaction F_(1,32)_ = 1.125, *p* = 0.2968; *Striatum*: sex F_(1,32)_ = 12.01, *p* = 0.0015; prenatal stress F_(1,32)_ = 7.435, *p* = 0.0103; treatment F_(1,32)_ = 1.032, *p* = 0.3174; sex vs. prenatal stress F_(1,32)_ = 0.01471, *p* = 0.9042; sex vs. treatment F_(1,32)_ = 4.078, *p* = 0.0519; prenatal stress vs. treatment F_(1,32)_ = 0.2664, *p* = 0.6093; interaction [F_(1,32)_ = 4.821, *p* = 0.0355]. No statistically significant differences were found between experimental groups regarding the levels of IL-6. Additional information on the effects of prenatal stress on IL-6 can be found in Supplementary Fig. 2.

**Fig. 4:**
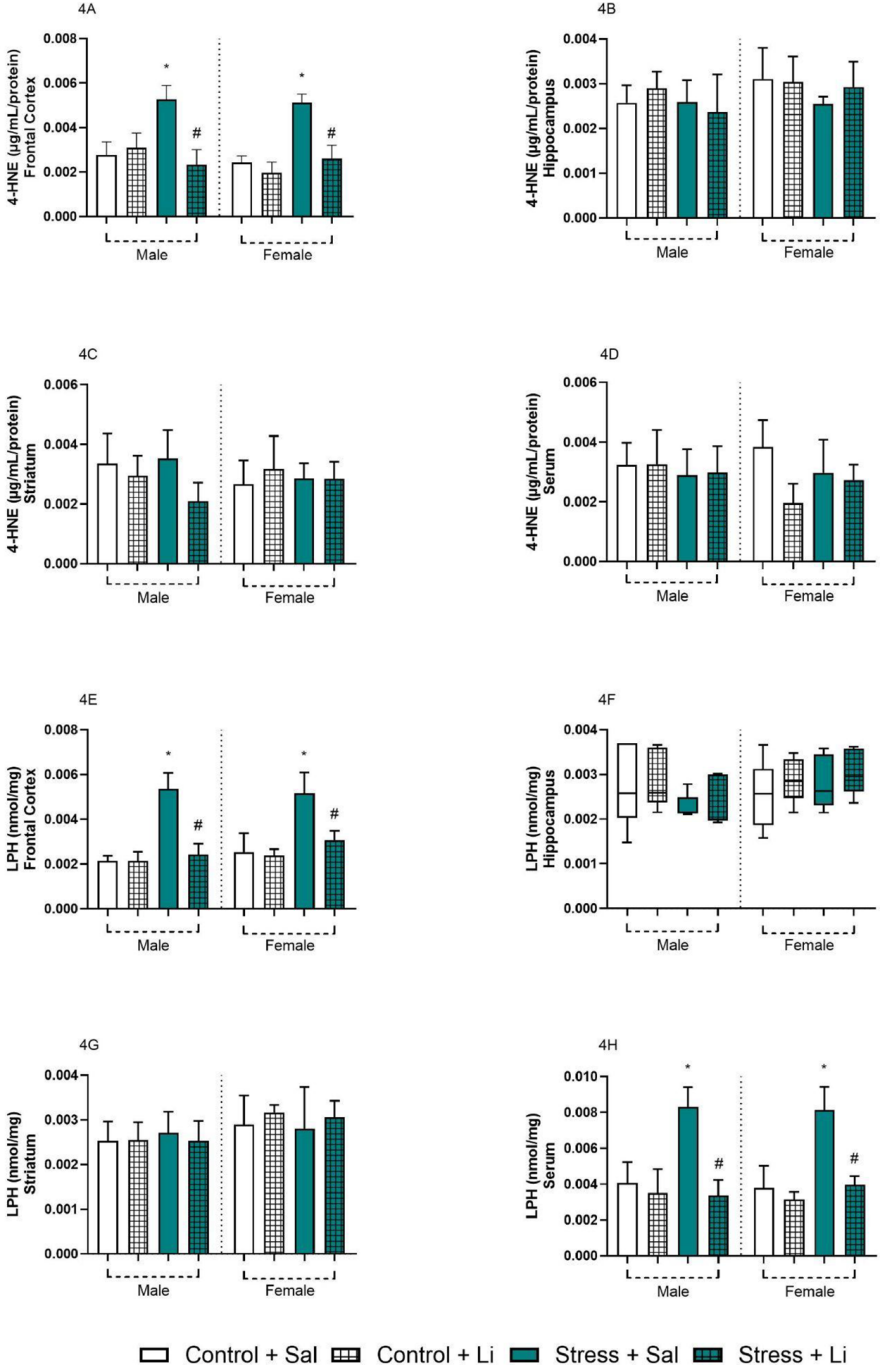
Data from the brain and serum levels of 4-hydroxynonenal (4-HNE) and lipid peroxidation activity of lipid hydroperoxide (LPH) of the offspring from dams exposed to prenatal stress and treated with lithium (Li) at adulthood (60 days old). Results are presented as columns, representing mean and standard deviation. Three-way ANOVA followed by Tukey’s post hoc test. *p < 0.05 as compared to Control + Sal of the same sex; #*p* < 0.05 as compared to Stress + Sal of the same sex

From the analysis of prenatal stress in the offspring brain, Fig. 4 represents the levels of 4-HNE and LPH, both markers of lipid peroxidation. Regarding the levels of 4-HNE in the frontal cortex, the three-way ANOVA indicated a significant difference in prenatal stress [F_(1,32)_ = 54.90, *p* < 0.0001], treatment [F_(1,32)_ = 66.74, *p* < 0.0001], and prenatal stress vs. treatment [F_(1,32)_ = 54.55, *p* < 0.0001]. No significant differences were found in the sex [F_(1,32)_ = 2.758, *p* = 0.1066], sex vs. prenatal stress [F_(1,32)_ = 4.053, *p* = 0.0526], sex vs. treatment [F_(1,32)_ = 0.09275, *p* = 0.7627], and the interaction [F_(1,32)_ = 2.328, *p* = 0.1369]. Post hoc analyses showed that, in both sexes, the levels of 4-HNE were increased in the frontal cortex of the offspring from stressed dams. Also, the effects of prenatal stress in the 4-HNE markers were reverted by Li administration in the adult offspring. On the hippocampus, striatum, and serum, no significant differences were observed according to ANOVA followed by Tukey’s post hoc [*Hippocampus*: sex F_(1,31)_ = 2.776, *p* = 0.1058 prenatal stress F_(1,31)_ = 2.776, *p* = 0.1058 treatment F_(1,31)_ = 0.3327, *p* = 0.5683 sex vs. prenatal stress F_(1,31)_ = 0.05153, *p* = 0.8219 sex vs. treatment F_(1,31)_ = 0.08316, *p* = 0.7750 prenatal stress vs. treatment F_(1,31)_ = 0.02357, *p* = 0.8790 interaction F_(1,31)_ = 1.889, *p* = 0.1792; *Striatum*: sex F_(1,32)_ = 0.1544, *p* = 0.6970 prenatal stress F_(1,32)_ = 0.6699, *p* = 0.4191 treatment F_(1,32)_ = 1.764, *p* = 0.1935 sex vs. prenatal stress F_(1,32)_ = 0.2791, *p* = 0.6009 sex vs. treatment F_(1,32)_ = 5.240, *p* = 0.0288 prenatal stress vs. treatment F_(1,32)_ = 2.276, *p* = 0.1412 interaction F_(1,32)_ = 0.2537, *p* = 0.6179; *Serum*: sex F_(1,32)_ = 0.6316, *p* = 0.4326 prenatal stress F_(1,32)_ = 0.4369, *p* = 0.5134 treatment F_(1,32)_ = 3.349, *p* = 0.0766 sex vs. prenatal stress F_(1,32)_ = 0.2309, *p* = 0.6341 sex vs. treatment F_(1,32)_ = 4.073, *p* = 0.0520 prenatal stress vs. treatment F_(1,32)_ = 2.388, *p* = 0.1321 interaction F_(1,32)_ = 2.002, *p* = 0.1668].

In addition, three-way ANOVA revealed significant differences in the LPH levels in the frontal cortex [sex F_(1,32)_ = 2.053, *p* = 0.1617 prenatal stress F_(1,32)_ = 83.19, *p* < 0.0001 treatment F_(1,32)_ = 48.03, *p* < 0.0001 sex vs. prenatal stress F_(1,32)_ = 0.08440, *p* = 0.7733 sex vs. treatment F_(1,32)_ = 0.8106, *p* = 0.3747 prenatal stress vs. treatment F_(1,32)_ = 42.30, *p* < 0.0001 interaction F_(1,32)_ = 1.704, *p* = 0.2011], and the serum [sex F_(1,32)_ = 0.03569, *p* = 0.8513 prenatal stress F_(1,32)_ = 49.34, *p* < 0.0001 treatment F_(1,32)_ = 60.91, *p* < 0.0001 sex vs. prenatal stress F_(1,32)_ = 0.6307, *p* = 0.4330] sex vs. treatment F_(1,32)_ = 0.2678, *p* = 0.6084 prenatal stress vs. treatment F_(1,32)_ = 35.86, *p* < 0.0001 interaction F_(1,32)_ = 0.4203, *p* = 0.5214]. In both samples, the levels of LPH were significantly increased in male and female offspring from stressed dams as compared to Control + Sal group. Also, Li administration reverted all these alterations. No significant differences were found in the hippocampus, according to Kruskal-Wallis [*H* = 6.553, *p* = 0.4768], and striatum, according to three-way ANOVA [sex F_(1,32)_ = 5.863, *p* = 0.0213 prenatal stress F_(1,32)_ = 0.001401, *p* = 0.9704 treatment F_(1,32)_ = 0.2799, *p* = 0.6004 sex vs. prenatal stress F_(1,32)_ = 0.2906, *p* = 0.5936 sex vs. treatment F_(1,32)_ = 1.089, *p* = 0.3045 prenatal stress vs. treatment F_(1,32)_ = 0.1169, *p* = 0.7346 interaction F_(1,32)_ = 0.07726, *p* = 0.7828].

**Fig. 5:**
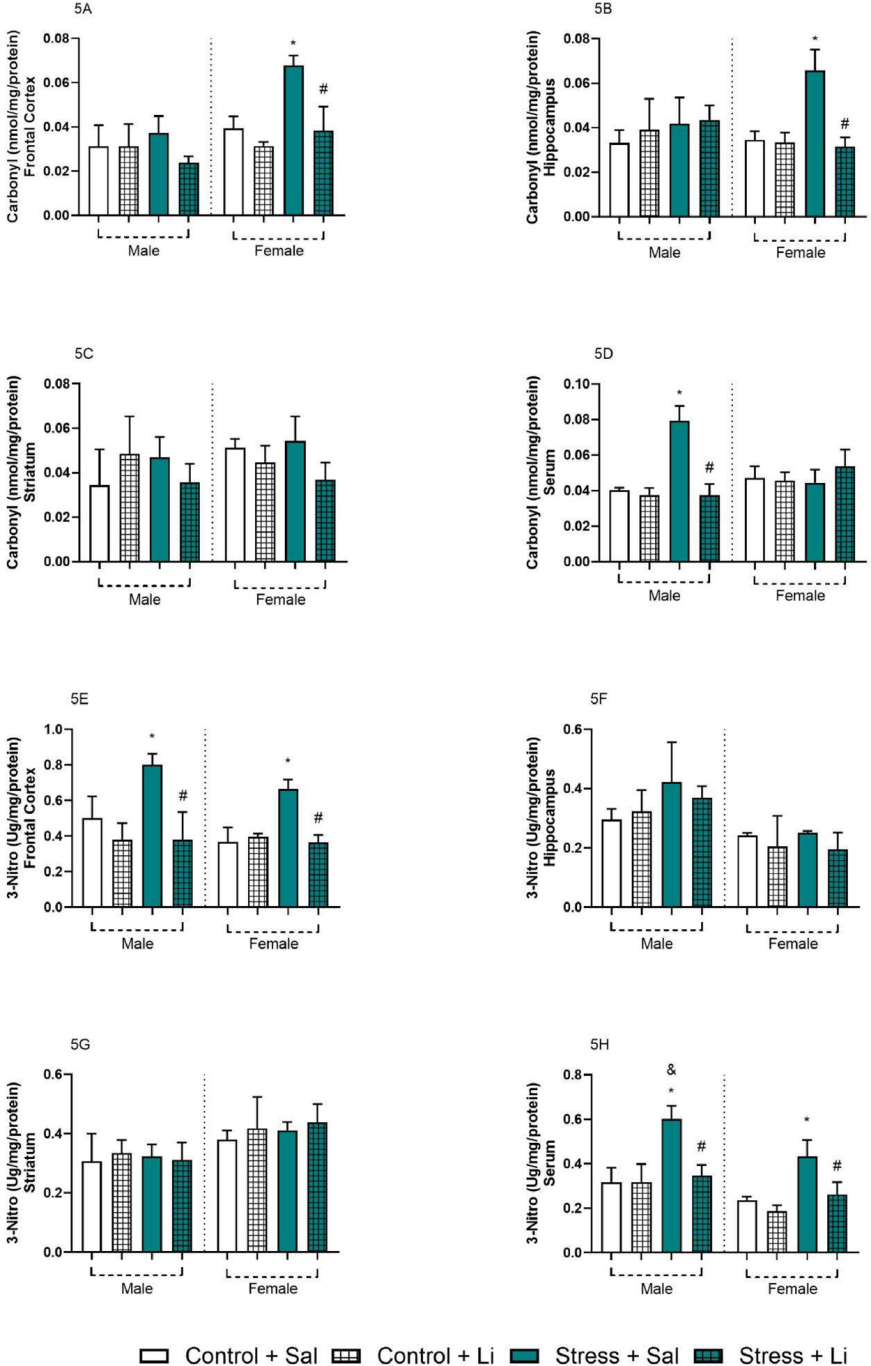
Brain and serum levels of carbonyl and 3-nitrotyrosine (3-Nitro) in the offspring exposed to prenatal stress and treated with lithium (Li) at adulthood (60 days old). Data are presented as columns representing mean and standard deviation of the mean. Three-way ANOVA followed by Tukey’s post hoc test. *p < 0.05 as compared to Control + Sal group of the same sex; #*p* < 0.05 as compared to Stress + Sal of the same sex; &*p* < 0.05 as compared to the same group of the opposite sex

Expanding the analysis on the effects of prenatal stress in oxidative/nitrosative stress markers, the levels of carbonyl and 3-Nitro are depicted in Fig. 5. On the carbonyl content in the frontal cortex, three-way ANOVA showed significant differences in sex F_(1,31)_ = 5.560, *p* = 0.0249, prenatal stress F_(1,31)_ = 24.3, *p* < 0.0001, treatment F_(1,31)_ = 49.68, *p* < 0.0001, *p* = 0.7906, sex vs. treatment F_(1,31)_ = 5.475, *p* = 0.0259, prenatal stress vs. treatment F_(1,31)_ = 29.92, *p* < 0.0001. No significant differences were detected in sex vs. prenatal stress F_(1,31)_ = 0.07174, and the interaction F_(1,31)_ = 0.04568, *p* = 0.8322. Tukey’s post hoc indicated that only the levels of carbonyl in the frontal cortex of stressed dams were significantly increased as compared to control. Li treatment reversed this alteration. Similarly, three-way ANOVA followed by Tukey’s post hoc found the same pattern of alteration in the female offspring [sex F_(1,32)_ = 0.5283, *p* = 0.4726 prenatal stress F_(1,32)_ = 16.44, *p* = 0.0003 treatment F_(1,32)_ = 7.015, *p* = 0.0124 sex vs. prenatal stress F_(1,32)_ = 2.485, *p* = 0.1248 sex vs. treatment F_(1,32)_ = 16.78, *p* = 0.0003 prenatal stress vs. treatment F_(1,32)_ = 12.44, *p* = 0.0013 interaction F_(1,32)_ = 7.547, *p* = 0.0098]. On the other hand, the opposite effect was found in the carbonyl serum levels. Three-way ANOVA demonstrated significant alterations in the prenatal stress F_(1,31)_ = 28.79, *p* < 0.0001, treatment F_(1,31)_ = 20.35, *p* < 0.0001, sex vs. prenatal stress F_(1,31)_ = 16.83, *p* = 0.0003, sex vs. treatment F_(1,31)_ = 40.51, *p* < 0.0001, prenatal stress vs. treatment F_(1,31)_ = 12.01, *p* = 0.0016, interaction F_(1,31)_ = 36.85, *p* < 0.0001. Post hoc analysis indicated that only male offspring from the prenatal Stress + Sal group presented increased serum levels of carbonyl as compared to control. Administration of Li reverted this alteration. No significant alterations were found in the striatum [sex F_(1,31)_ = 2.484, *p* = 0.1252, prenatal stress F_(1,31)_ = 0.1339, *p* = 0.7169, treatment F_(1,31)_ = 2.323, *p* = 0.1376, sex vs. prenatal stress F_(1,31)_ = 0.1082, *p* = 0.7444, sex vs. treatment F_(1,31)_ = 3.846, *p* = 0.0589, prenatal stress vs. treatment F_(1,31)_ = 6.965, *p* = 0.0129, interaction F_(1,31)_ = 1.131, *p* = 0.2958].

Regarding the levels of 3-Nitro on the frontal cortex, three-way ANOVA demonstrated significant differences in sex F_(1,31)_ = 5.560, *p* = 0.0249, prenatal stress F_(1,31)_ = 24.30, *p* < 0.0001, treatment F_(1,31)_ = 49.68, *p* < 0.0001, sex vs. treatment F_(1,31)_ = 49.68, *p* < 0.0001, and prenatal stress vs. treatment F_(1,31)_ = 29.92, *p* < 0.0001. No significant differences were detected in sex vs. prenatal stress F_(1,31)_ = 0.07174, *p* = 0.7906, and the interaction F_(1,31)_ = 0.04568, *p* = 0.8322. Tukey’s post hoc analysis showed that, in both sexes, prenatal stress increased the levels of 3-Nitro in the frontal cortex, and that Li reverted these alterations. Similarly, three-way ANOVA revealed, in the serum, a statistically significant difference in sex F_(1,32)_ = 41.44, *p* < 0.0001, prenatal stress F_(1,32)_ = 67.09, *p* < 0.0001, treatment F_(1,32)_ = 43.33, *p* < 0.0001, prenatal stress vs. treatment F_(1,32)_ = 27.80, *p* < 0.0001. No statistical differences were found in the sex vs. prenatal stress F_(1,32)_ = 0.3186, *p* = 0.5764, sex vs. treatment F_(1,32)_ = 0.1954, *p* = 0.6614, and the interaction F_(1,32)_ = 3.442, *p* = 0.0728. Post hoc test in the serum samples revealed that, in both sexes, the levels of 3-Nitro were higher in the offspring from stressed dams, as compared to control. Treatment with Li reverted all alterations. Additionally, the serum levels of 3-Nitro were higher in males in the Stress + Sal groups as compared to females in the same group. No significant differences were found in the hippocampus and striatum levels of 3-Nitro according to three-way ANOVA followed by Tukey’s post hoc test [Hippocampus: sex F_(1,30)_ = 29.01, *p* < 0.0001 prenatal stress F_(1,30)_ = 3.201, *p* = 0.0837 treatment F_(1,30)_ = 1.518, *p* = 0.2275 sex vs. prenatal stress F_(1,30)_ = 3.295, *p* = 0.0795 sex vs. treatment F_(1,30)_ = 0.5073, *p* = 0.4818 prenatal stress vs. treatment F_(1,30)_ = 1.039 *p* = 0.3163 interaction F_(1,30)_ = 0.4092, *p* = 0.5272; Striatum: sex F_(1,32)_ = 21.72, *p* < 0.0001 prenatal stress F_(1,32)_ = 0.3453, *p* = 0.5609 treatment F_(1,32)_ = 0.9737, *p* = 0.3312 sex vs. prenatal stress F_(1,32)_ = 0.5030, *p* = 0.4833 sex vs. treatment F_(1,32)_ = 0.3955, *p* = 0.5339 prenatal stress vs. treatment F_(1,32)_ = 0.3729, *p* = 0.5457 interaction F_(1,32)_ = 0.1318, *p* = 0.7190]. No differences were found regarding the activity of GPx and GR. Additional information can be found in Supplementary Figs. 3 and 4.

Regarding the biochemical analysis of the dams (Table 1), HPA axis markers indicated no statistically significant differences between stressed and control groups [ACTH: *t* = 1.098, df = 8, *p* = 0.3043; Corticosterone: *t* = 0.3867, df = 8, *p* = 0.7091]. In the context of the inflammatory markers, IL-1ꞵ level was reduced in the frontal cortex of stressed dams [*t* = 4.393, df = 7, *p* = 0.0032]. TNF-ɑ levels were increased in the serum [*t* = 5.116, df = 8, *p* = 0.0009], and hippocampus [*t* = 2.308, df = 8, *p* = 0.0499]. In the serum, stressed dams presented higher levels of 4-HNE [*t* = 5.935, df = 8, *p* = 0.0003], LPH [*t* = 5.937, df = 7, *p* = 0.0006], carbonyl [*t* = 9.926, df = 8, *p* < 0.0001], and 3-Nitro [*t* = 9.602, df = 8, *p* < 0.0001]. Lastly, the dams subjected to stress presented lower levels of 3-Nitro in the frontal cortex [*U* = 0, *p* = 0.0079], and a lower GPx activity in the striatum [*t* = 2.363, df = 8, *p* = 0.0458].

Table 1 presents data provided by the biochemical analyses of the dams.

Data from the analysis of the hypothalamic-pituitary-adrenal (HPA) axis, oxidative stress, and inflammation parameters in the frontal cortex, hippocampus, striatum, and serum of control and stressed dams. Parametric data are presented as mean ± standard deviation of the mean (SD) evaluated with the Student’s *t*-test. Nonparametric data are presented as median (CI) evaluated with the Mann-Whitney *U* test. HPA-axis data are presented in pg/mL. Inflammation markers in the brain structures are presented in pg/100 mg tissue and serum in pg/mL. Oxidative stress markers are presented as follows: 4-hydroxinonenal (4-HNE): ng/mL/protein; lipid peroxidation activity of lipid hydroperoxide (LPH): nmol/mg protein; carbonyl content: nmol/mg protein; 3-nitrotyrosine (3-Nitro): ug/mg protein; glutathione peroxidase (GPx) and glutathione reductase (GR): nmol/min/mL. * *p* ≤ 0.05 as compared to the control group.

## Discussion

The present study aimed to evaluate the effects of prenatal stress on the behavior, HPA axis, inflammation, and oxidative stress in both dams and their offspring while also examining the impact of Li treatment. Partially consistent with our first hypothesis, we found that prenatal stress induced significant changes in various offspring behavioral parameters, stress responses, inflammatory markers, and oxidative damage, with differences observed according to sex. Furthermore, the findings partially corroborated our second hypothesis, demonstrating Li potential to reverse the effects of prenatal stress, though with distinct responses between males and females, and no effect on the hyperactivity observed in the female offspring.

The literature suggests that maternal stress can lead to poor maternal care, a potential mechanism affecting the offspring brain development and behavior [40–42]. In the present study, no significant differences were found in the overall maternal care index between groups, except in the ABN3 position, where we observed a reduction in the group subjected to stress as compared to the control. The ABN3 position, characterized by a fully arched back during nursing, requires greater energy expenditure from the dam. Previous studies have demonstrated that maternal separation models are associated with poor maternal care [42, 43]. However, there is no consensus regarding the effects of prenatal stress on maternal care, with some studies reporting increased maternal care and others indicating disorganized postpartum care [44–47]. These findings suggest that prenatal stress and postnatal adversity can independently and cumulatively impair maternal behavior, potentially affecting maternal attention, emotional regulation, and motivation. Also, one other explanation for alteration in the ABN3 position is due to maternal exhaustion rather than altered care quality. However, more studies would be necessary to further analyze this possibility. Overall, we hypothesize that there were no significant alterations between the stressed and control groups, since when all day-to-day maternal care behaviors were combined and analyzed as a group only one parameter showed significant differences.

The effects of prenatal stress on adult offspring behavior revealed significant sex differences. In females, prenatal stress increased the number of crossings and rearings, indicative of hyperactive behavior, which Li failed to reverse. In males, prenatal stress increased the latency duration in the open field test, reflecting anxiety-like behavior, which was reversed by Li treatment. Studies indicate that male and female offspring respond differently to prenatal stress, which may partly explain these sex-dependent behavioral changes [48, 49]. Mechanisms modulating these responses include hormonal and epigenetic influences during the intrauterine period [29, 48, 49]. Furthermore, as only female offspring exhibited hyperactivity, they may be more vulnerable to this response type [50]. Additionally, since the female offspring were not stage-matched regarding their estrous cycle this could have partially influenced the results. Also, considering a translational approach, clinical trials do not control for the menstrual cycle of female individual Further studies should focus on understating the role of estrous cycle changes in the offspring of stressed dams.

Li is widely recognized as the gold-standard treatment for bipolar disorder, particularly for managing manic episodes. Thus, it was anticipated that Li would reverse the hyperactivity observed in females in the prenatal stress group. However, the lack of response suggests this behavior may not reflect a mania-like condition, often modeled in rodents as an analog for bipolar mania [35, 51]. Instead, this hyperactivity might align more with schizophrenia-associated behaviors, given its association with dysfunctions in dopaminergic processing in schizophrenia models [52–54]. We reinforce that this is only an initial hypothesis of the behavior alterations found herein and to validate this hypothesis, future studies should explore schizophrenia-like behaviors, such as deficits in the prepulse inhibition [55], and evaluate antipsychotic treatments. Another interpretation is that this hyperactivity represents a model of Li-resistant mania, reflecting a condition common in bipolar patients [56, 57]. Similarly to the schizophrenia-associated behaviors hypothesis, this interpretation of the Li-resistant mania is an initial hypothesis, and further studies on different aspects of mania-like behavior are needed to validate this theory. On the other hand, in male offspring, Li treatment reversed the increased latency, suggesting an anxiolytic-like effect. Corroborating this, a recent study demonstrated that Li monotherapy significantly improved anxiety symptoms in patients with bipolar disorder [58]. These findings underscore Li anxiolytic potential and highlight the need for further research to understand the underlying neurobiological mechanisms and clinical applications for anxiety management.

We found that prenatal stress led to hyperactivation of the HPA axis in the offspring. This was evidenced by elevated levels of ACTH and corticosterone, with a more pronounced increase in ACTH levels observed in females as compared to males. These differences in stress responses may be linked to the behavioral variations observed between sexes. Alterations in the HPA axis have consistently been associated to behavioral changes characteristic of depression and bipolar disorder [59]. In humans, sex differences in HPA axis responses have also been documented. Contrary to the findings of this study, previous research has shown higher ACTH levels in young men as compared to young women, while cortisol levels were similar in both sexes [60, 61]. These discrepancies have been attributed to a potential relative resistance to ACTH in men, requiring higher levels of this hormone to achieve the same adrenal responsiveness observed in women. The literature frequently highlights divergences in sex-related influences on the HPA axis across species, emphasizing the need for further studies to better understand these differences. Another factor that may impact the HPA axis response and female behavior is estradiol and its receptor activity, such as ERα and ERβ [62, 63]. However, this study did not assess estradiol levels or the estrous cycle in female rats.

As expected, Li treatment effectively reversed the HPA axis alterations caused by prenatal stress in both sexes. Previous studies in animal models of mania and bipolar disorder have demonstrated this therapeutic effect of the drug [35, 36]. However, the precise mechanism by which Li modulates the HPA axis remains unclear. One theory regarding the HPA axis’s role in the pathophysiology of mental disorders suggests that glucocorticoid receptor imbalances occur in various brain regions [23, 64–66]. This imbalance, however, may originate in intrauterine development. Since chronic unpredictable stress is known to hyperactivate the HPA axis, this excessive production of glucocorticoids may cross the placental barrier and influence the fine regulation of this endocrine system [67]. Despite this association, in this study, the levels of ACTH and corticosterone assessed in the dams did not show significant differences between groups. This finding may be explained by the protective influence of oxytocin, which has stress-regulating and neuroprotective properties that may mitigate the effects of prenatal HPA axis hyperactivation [68–71]. Thus, the absence of changes in the maternal hormone levels could reflect oxytocin’s influence during the postpartum period, as the assessments were conducted after birth and weaning (postnatal day 21).

Regarding inflammatory parameters, prenatal stress reduced serum IL-1β levels in male offspring while increasing TNF-α levels in the frontal cortex and serum in both sexes. In dams, prenatal stress increased IL-1β levels in the frontal cortex and TNF-α levels in the hippocampus. The reduction or absence of changes in the levels of the pro-inflammatory cytokine IL-1β in offspring, depending on sex and tissue analyzed, reinforces this interpretation. Additionally, the levels of IL-6 in the offspring were unchanged by the prenatal stress and the treatment with Li. These findings suggest that in both offspring and dams, the inflammatory response occurs in a chronic stage, characterized by the coexistence of pro- (increase levels of TNF-α) and anti-inflammatory (unchanged levels of IL-6 and reduction of IL-1β) mechanisms [72]. In the case of dams, the reduction of IL-1β in the frontal cortex after the stress protocol also suggests the presence of compensatory mechanisms, probably activated by the long-time interval between the stress protocol and the assessment of the inflammatory profile. However, this reduction does not imply the absence of inflammation or oxidative stress, either in mothers or offspring.

Structural damage in the brains of prenatally stressed offspring is partly due to oxidative stress [73]. Here, prenatal stress elevated 4-HNE levels in the frontal cortex of the offspring and increased LPH levels in the frontal cortex and serum in both sexes. Additionally, it increased carbonyl levels in the frontal cortex and hippocampus of females, whereas in males, these increases were observed in the frontal cortex and serum. These findings suggest that, despite the lack of widespread pro-inflammatory responses at the time of evaluation, oxidative and inflammatory impairments persisted in the brain. Interestingly, similar alterations were observed in dams, but only in serum. Alteration in frontal cortex and hippocampus are a stable of different psychiatric such as bipolar disorder, schizophrenia, and depression [24–75]. Also, alteration on the oxidative balance in the hippocampus is related to neurodegeneration diseases [76]. Taking together, these results indicate that chronic low-grade inflammation accompanies oxidative stress in both dams and offspring.

As anticipated, Li treatment reversed all inflammatory and oxidative changes induced by prenatal stress in the offspring, demonstrating its anti-inflammatory and antioxidant properties. While the exact mechanisms remain unclear, Li likely exerts these effects by inhibiting glycogen synthase kinase 3β [77]. Furthermore, previous studies in animal models of mood disorders have also highlighted Li anti-inflammatory and antioxidant effects [77, 78]. However, these effects were not able to reverse the behavioral changes, suggesting the possible involvement of other underlying mechanisms. An alternative explanation is that the long-term effects of inflammation and oxidative stress on the neurodevelopment of the offspring caused irreversible behavioral changes that could not be corrected by short-term treatment.

Regarding the implications of the present study, the observed neurochemical changes suggest underlying mechanisms by which prenatal stress may increase vulnerability to psychiatric disorders. It is important to highlight that chronic low-grade inflammation is a common pathophysiological feature in many mood and stress-related disorders [25, 79, 80]. This condition is linked to long-term consequences, including neurodegeneration and cognitive deficits [81, 82]. Notably, the results of this study indicate that the inflammatory state persisted even several days after exposure to prenatal stress, highlighting its potential impact on the offspring development. One hypothesis for this developmental alteration is that the maternal inflammatory state may elicit an inflammatory response in the placenta, triggering epigenetic modifications, microglial activation, neuroinflammation, and oxidative stress [19, 20]. The data presented here support this hypothesis, demonstrating the presence of chronic low-grade inflammation accompanied by oxidative stress in both dams and their offspring. Given that these processes can significantly affect offspring development [81, 82], it is critical to explore interventions aimed at reducing inflammation and oxidative stress. Treatments targeting these mechanisms could help mitigate the damage, slow neuroprogression, and prevent the onset of mental disorders associated with prenatal stress. One limitation of this study is the use of only one behavioral measure. Further research is needed to evaluate additional behavioral changes induced by prenatal stress.

Some limitations of the results are important to be mentioned: (1) The effects of individual litters in the behavior and biochemical analysis could have influenced the results; to mitigate the effects all the animals were randomly divided into the treatment groups. (2) A single behavior test is insufficient to determine all the behavior outcomes induced by prenatal stress; more studies are needed.

In conclusion, the prenatal stress protocol caused long-lasting changes in behavior, inflammatory markers and oxidative stress, and HPA axis dysfunction in the offspring. Behavioral responses to prenatal stress varied between sexes, suggesting distinct mechanisms throughout development. Although Li reversed all inflammatory, oxidative, and HPA axis dysfunction alterations, it was not effective in correcting behavioral changes, raising the hypothesis of an animal model of Li-resistant mania or schizophrenia. Further studies are needed to fully elucidate these possibilities.

## Supplementary Information

Below is the link to the electronic supplementary material.

Supplementary Material 1

## Data Availability

The datasets generated during and/or analyzed during the current study are not publicly available but are available from the corresponding author on reasonable request.
