# Supplementary material for: Prenatal Stress Induces Changes in Behavior, HPA Axis, Inflammation, and Oxidative Stress in Adult Rats Offspring
Source: Neurochem Res. 2026 Feb 28;51(2):91. doi: 10.1007/s11064-026-04672-3 (PMC12950096; doi:10.1007/s11064-026-04672-3)
Supplement: Supplementary file 1 — Supplementary Material 1 [file 11064_2026_4672_MOESM1_ESM.docx]

**PRENATAL STRESS INDUCES CHANGES IN BEHAVIOR, HPA AXIS, INFLAMMATION, AND OXIDATIVE STRESS IN ADULT RATS OFFSPRING**

Jorge M. Aguiar-Geraldoa, Jefté Peper-Nascimentoa, José Henrique Cararoa, Taise Possamai-Dellaa, Alexandra I. Zugnoa, Anilkumar Pillaib,c; João Quevedod, Samira S. Valvassoria*

aTranslational Psychiatry Laboratory, Graduate Program in Health Sciences, University of Southern Santa Catarina (UNESC), Criciúma, SC, Brazil.

bCenter of Excellence on Mood Disorders, Faillace Department of Psychiatry and Behavioral Sciences, McGovern Medical School, The University of Texas Health Science Center at Houston (UTHealth), Houston, TX, USA; Neuroscience Graduate Program, The University of Texas MD Anderson Cancer Center UTHealth Graduate School of Biomedical Sciences, Houston, TX, USA; Translational Psychiatry Program, Faillace Department of Psychiatry and Behavioral Sciences, McGovern Medical School, The University of Texas Health Science Center at Houston (UTHealth), Houston, TX, USA.

cCharlie Norwood VA Medical Center, Augusta, GA, USA.

dCenter for Interventional Psychiatry, Faillace Department of Psychiatry and Behavioral Sciences, McGovern Medical School, The University of Texas Health Science Center at Houston (UTHealth Houston), Houston, TX, USA.

**Supplementary Figure 1** – Maternal Care

**Supplementary Figure 1** demonstrates maternal care with the offspring evaluations on the 1 to the 6 PND on the stressed and control dams. Accompanied by the day-to-day value of each parameter, a graph representing the sum of all six days was added. In mothers from the control group, the number of passive nursing (PND 6), ABN2 (PND 1 and 2), ABN3 (PND 2, 3, and 5), non-nursing contact (PND 3), and the total number of nursing and other behavior with the offspring (PND 2) were higher than in those mothers from the stressed group. In addition, the stressed group presented a higher number of blanked nursing (PND 3), carrying the offspring to the nest (PND 2), and the number of times that the mother remained away from the offspring (PND 2) when compared to the control group. Notably, when observing the sum of results obtained, only ABN3 was significantly decreased in the stress group.

Number of passive nursing Day 1 [U=91 ;p>0.9999] Day 2 [U=72 ;p= 0.3659] Day 3 [U= 89;p= 0.9332] Day 4 [U= 88;p= 0.8952] Day 5 [U= 60;p= 0.1367] Day 6 [U= 39;p= 0.0101] Total [U= 57.50;p= 0.1072]. Number Blanket nursing Day 1 [U= 73;p= 0.3941] Day 2 [U= 84;p= 0.7457] Day 3 [U= 46.50;p= 0.0284] Day 4 [U= 83;p= 0.7091] Day 5 [U= 68.50;p= 0.2827] Day 6 [U= 51;p= 0.0515] Total [U= 62.50;p= 0.1729]. Number of ABN1 Day 1 [U= 72;p= 0.3685] Day 2 [U= 67;p= 0.2525] Day 3 [U= 79;p= 0.5731] Day 4 [U= 81;p= 0.6379] Day 5 [U= 58;p= 0.1118] Day 6 [U= 90;p= 0.9712] Total [t=1.165, df=25 ;p=0.2549]. Number of ABN2 Day 1 [U= 39;p=0.0101 ] Day 2 [U= 43;p= 0.0184] Day 3 [U= 69;p= 0.2950] Day 4 [U= 56;p= 0.0909] Day 5 [U= 87.50;p= 0.8763] Day 6 [U= 66;p= 0.2327] Total [t=2.054, df=25; p= 0.0506]. Number of ABN3 Day 1 [U= 60;p= 0.1318] Day 2 [U= 45;p= 0.0223] Day 3 [U= 44.50;p= 0.0216] Day 4 [U= 73;p= 0.3894] Day 5 [U= 43;p= 0.0143] Day 6 [U= 59;p= 0.1201] Total [U= 42;p= 0.0160]. Number of nun-nursing contact Day 1 [U= 81;p= 0.6399] Day 2 [U= 79;p= 0.5700] Day 3 [U= 41.50;p= 0.0147] Day 4 [U= 74;p= 0.4185] Day 5 [U= 81.50;p= 0.6565] Day 6 [U= 60;p= 0.1350] Total [U= 86.50;p= 0.8391]. Number of cleaning Day 1 [U= 59.50;p= 0.1297] Day 2 [U= 65;p= 0.2053] Day 3 [U= 69.50;p= 0.3037] Day 4 [U= 70;p= 0.2996] Day 5 [U= 78;p= 0.5362] Day 6 [U= 89;p= 0.9317] Total [U= 17;p= 0.9004]. Carrying to the nest Day 1 [U= 78;p= 0.5281] Day 2 [U= 63;p= 0.0407] Day 3 [U= 71;p= 0.2966] Day 4 [U=84.50 ;p>0.9999] Day 5 [U= 78;p= 0.4815] Day 6 [U= 78;p= 0.4815] Total [U= 78;p= 0.5009]. Total number of nursing and contact Day 1 [U= 53.50;p= 0.0652] Day 2 [U= 37;p= 0.0072] Day 3 [U= 55;p= 0.0822] Day 4 [U= 62.50;p= 0.1718] Day 5 [U= 88;p= 0.8953] Day 6 [U= 67;p= 0.2495] Total [t=1.802, df=25 ;p= 0.0836]. Away from the offspring Day 1 [U= 53;p= 0.0618] Day 2 [U= 35;p= 0.0050] Day 3 [U= 55;p= 0.0822] Day 4 [U= 63.50;p= 0.1875] Day 5 [U= 88;p= 0.8953] Day 6 [U= 67;p= 0.2495] Total [t=1.832, df=25;p= 0.0789].


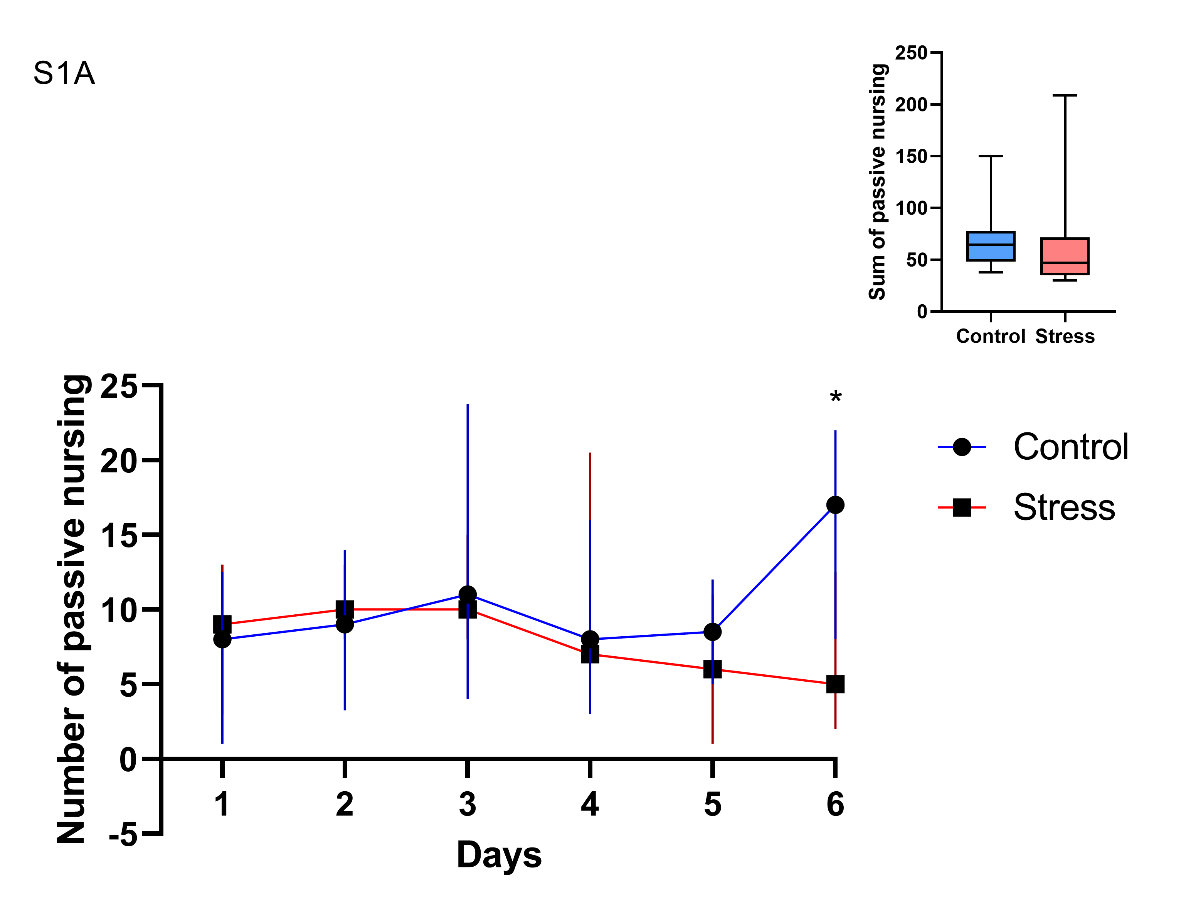


**
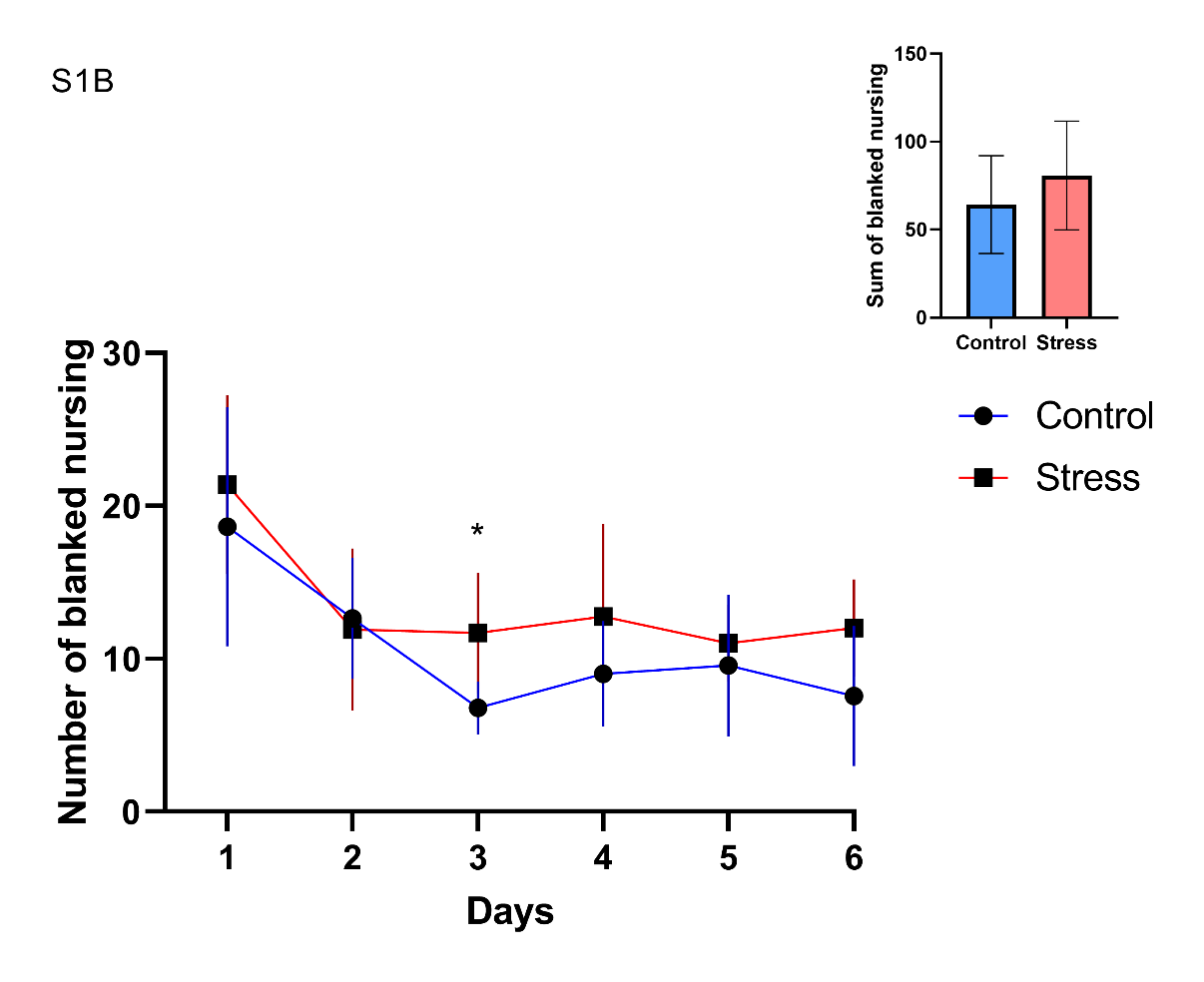
**

**
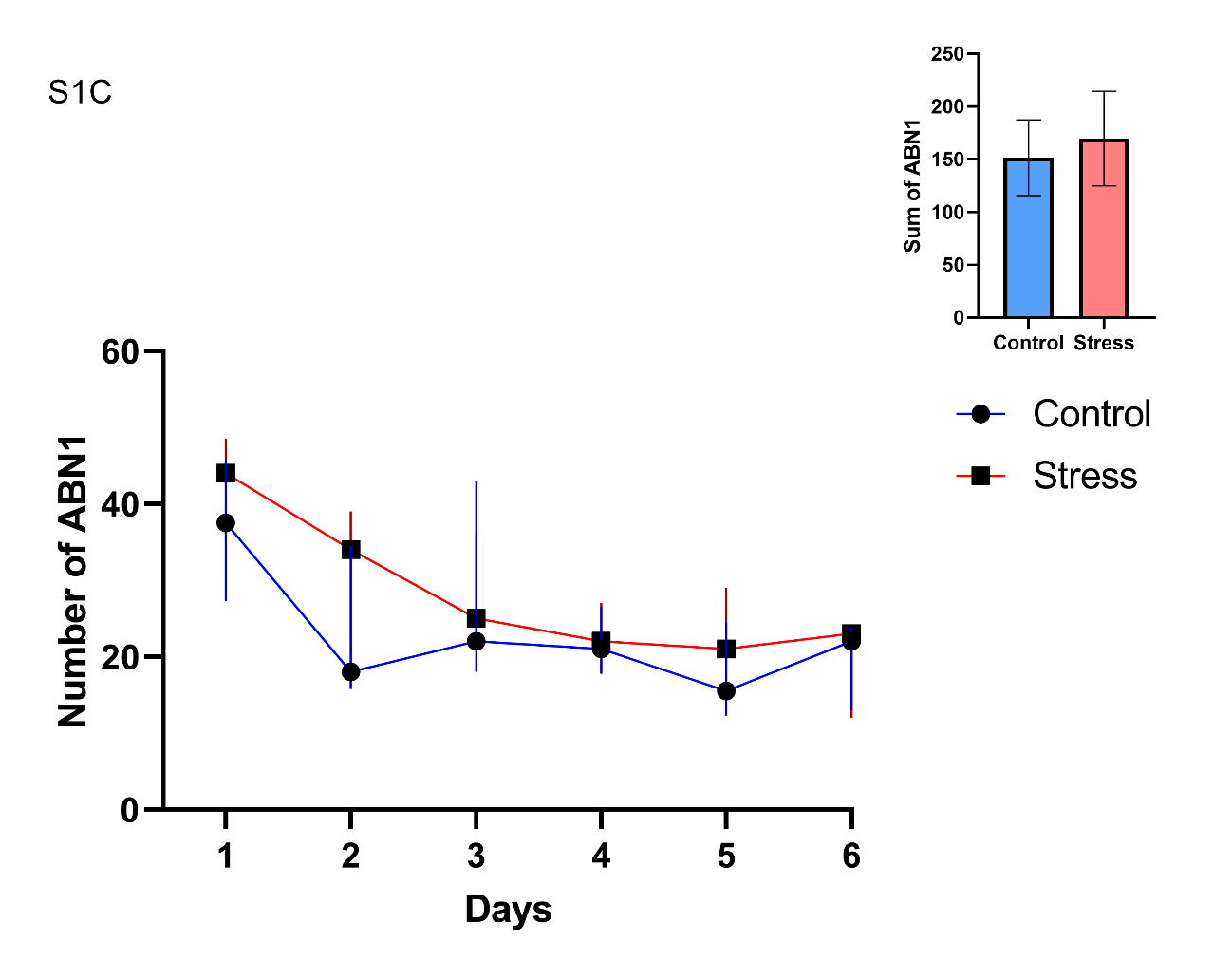
**

**
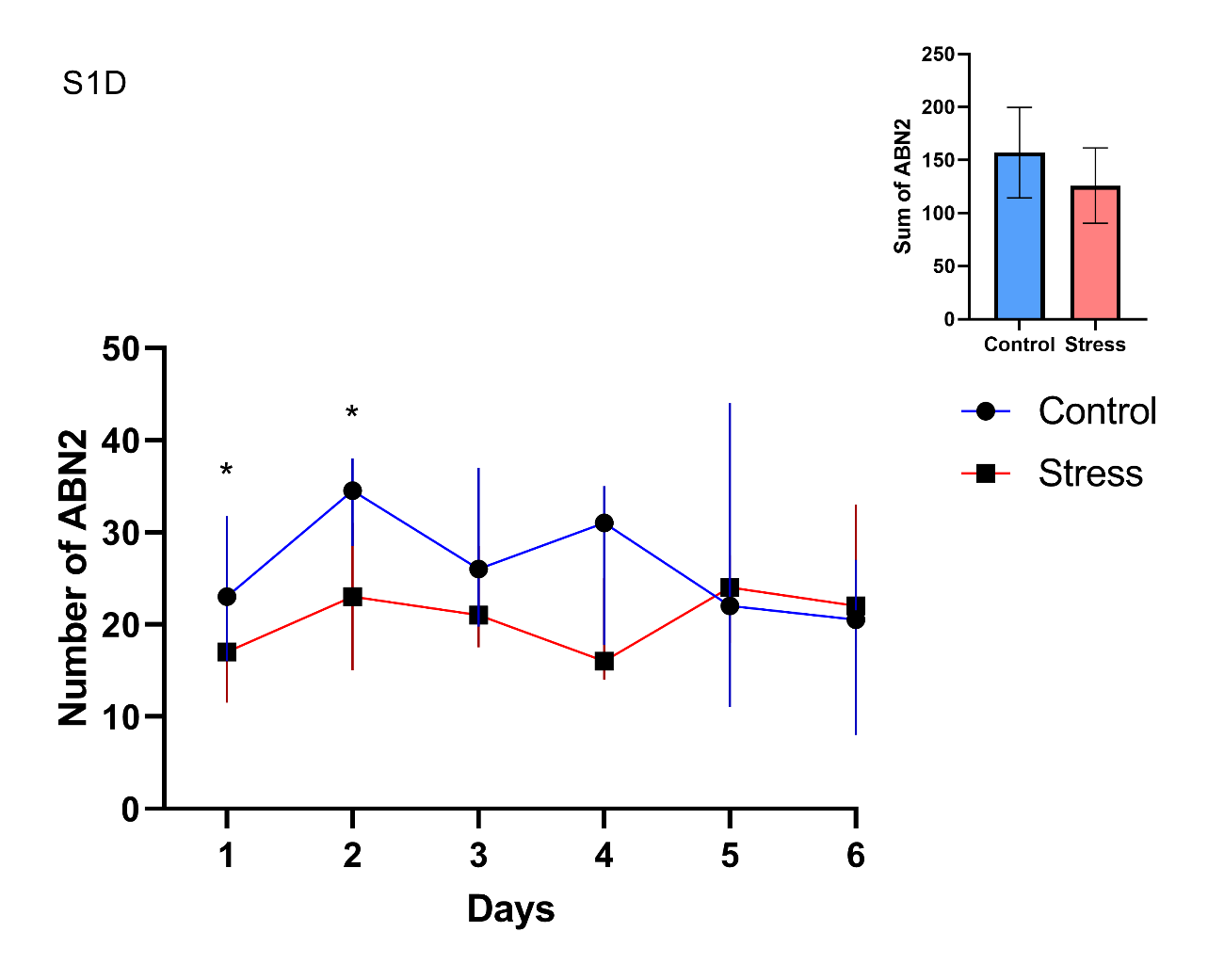
**

**
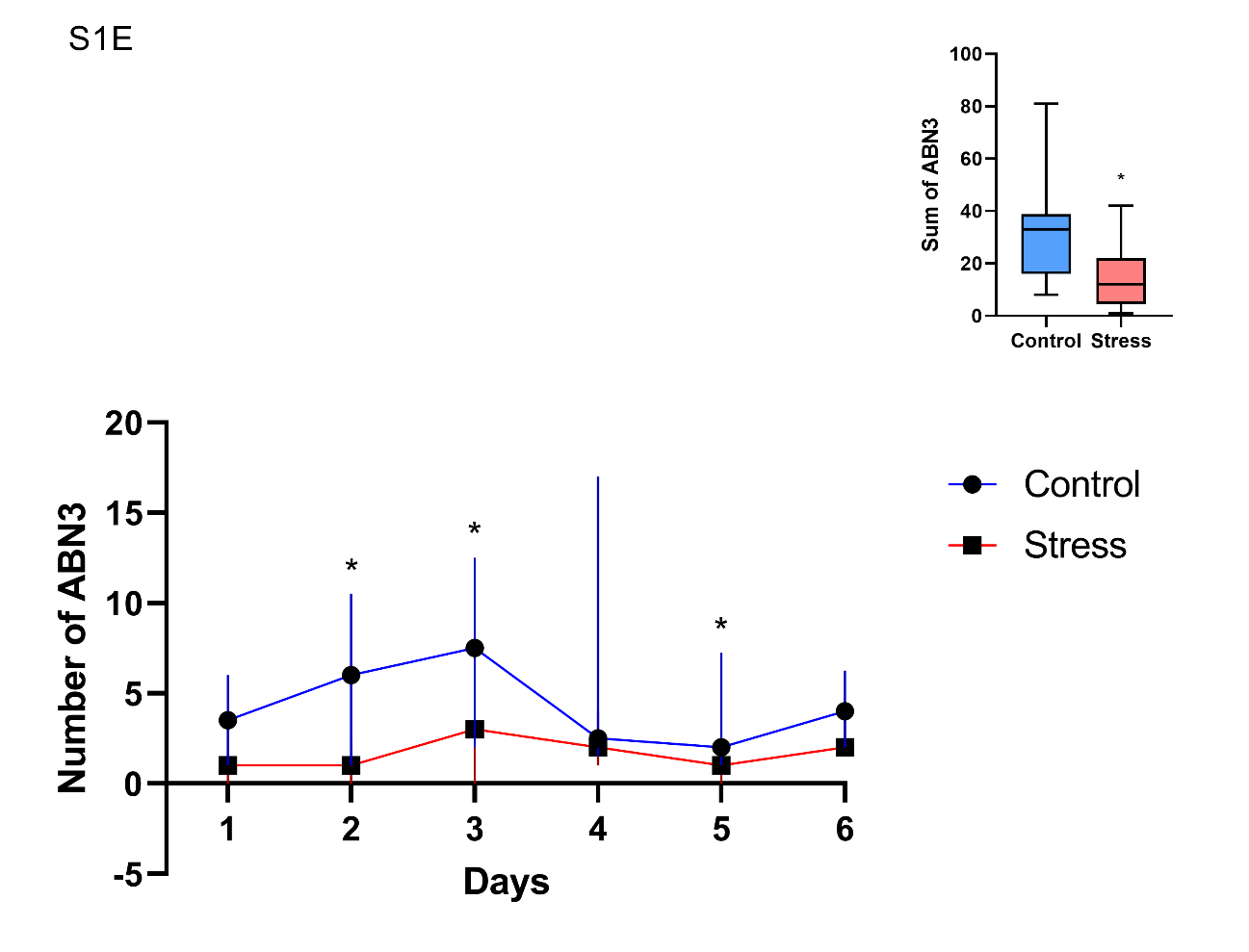
**

**
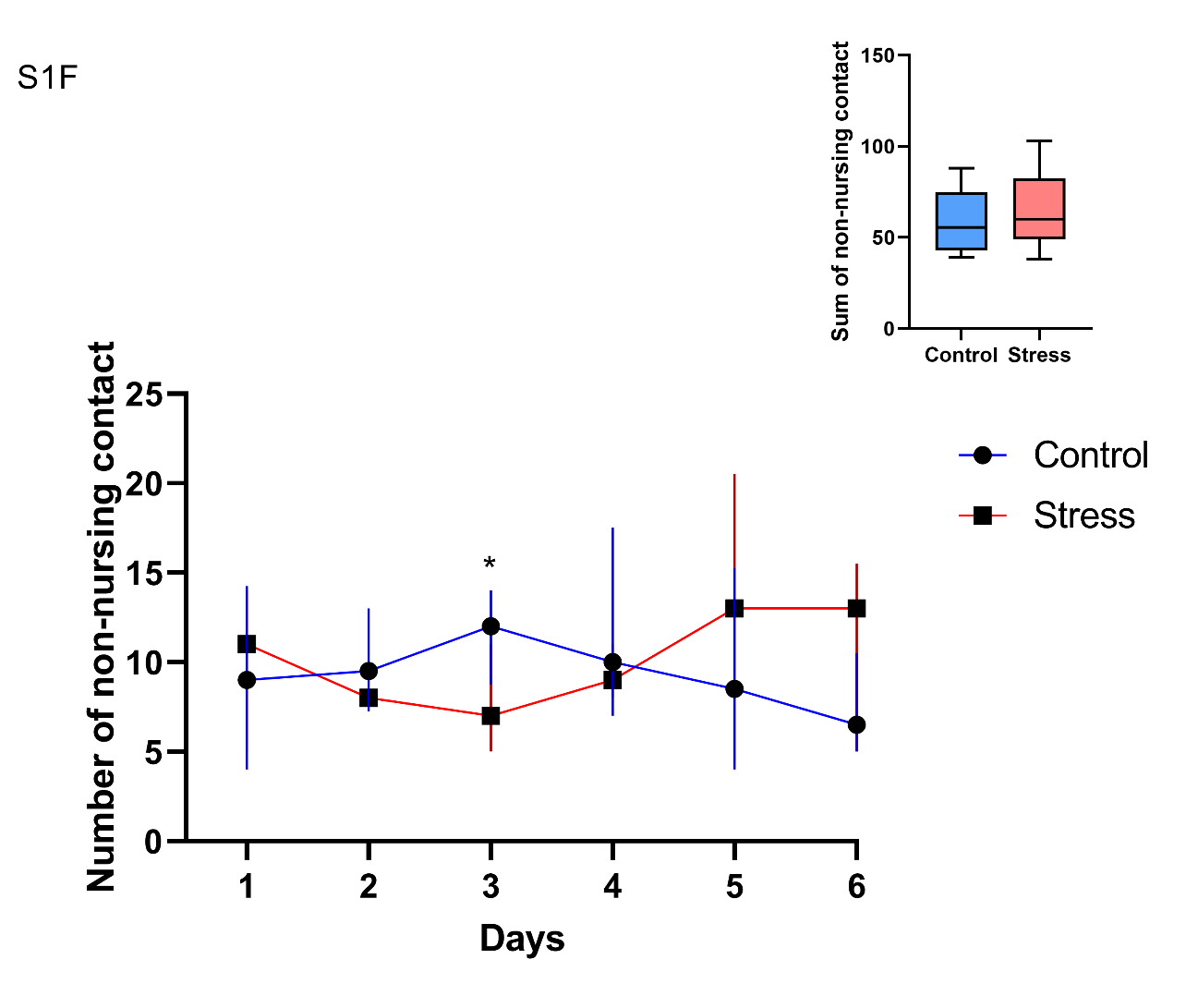
**

**
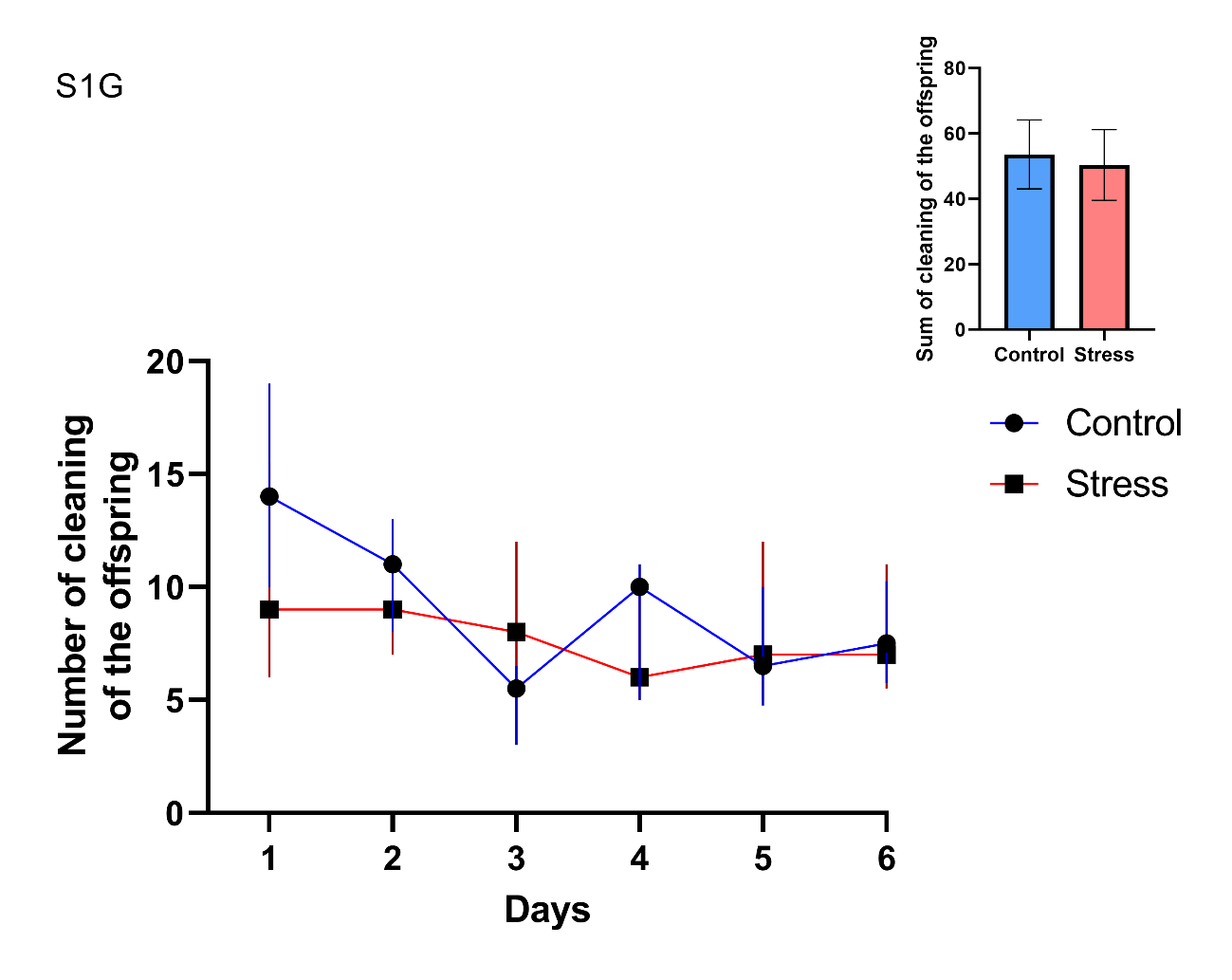
**

**
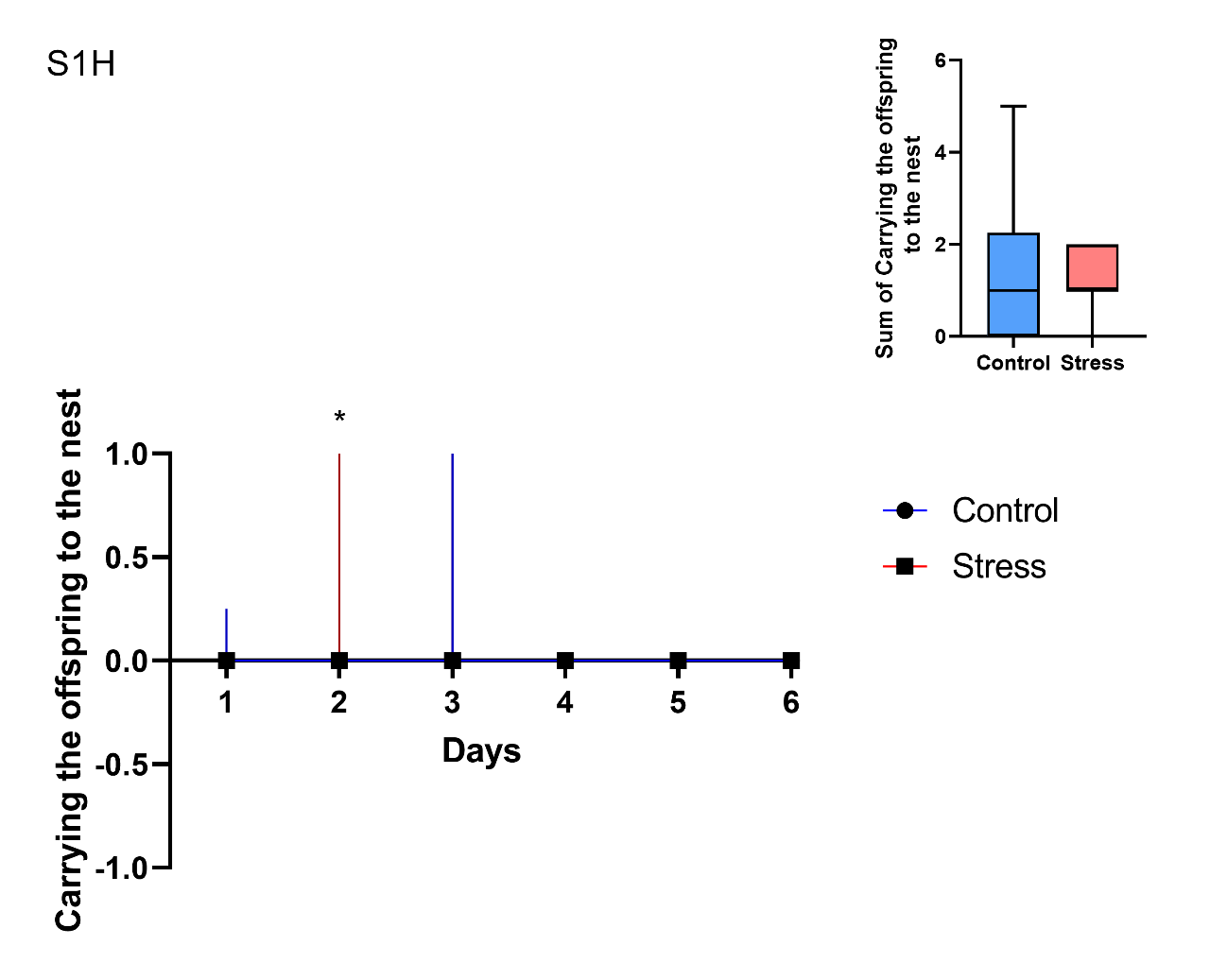
**

**
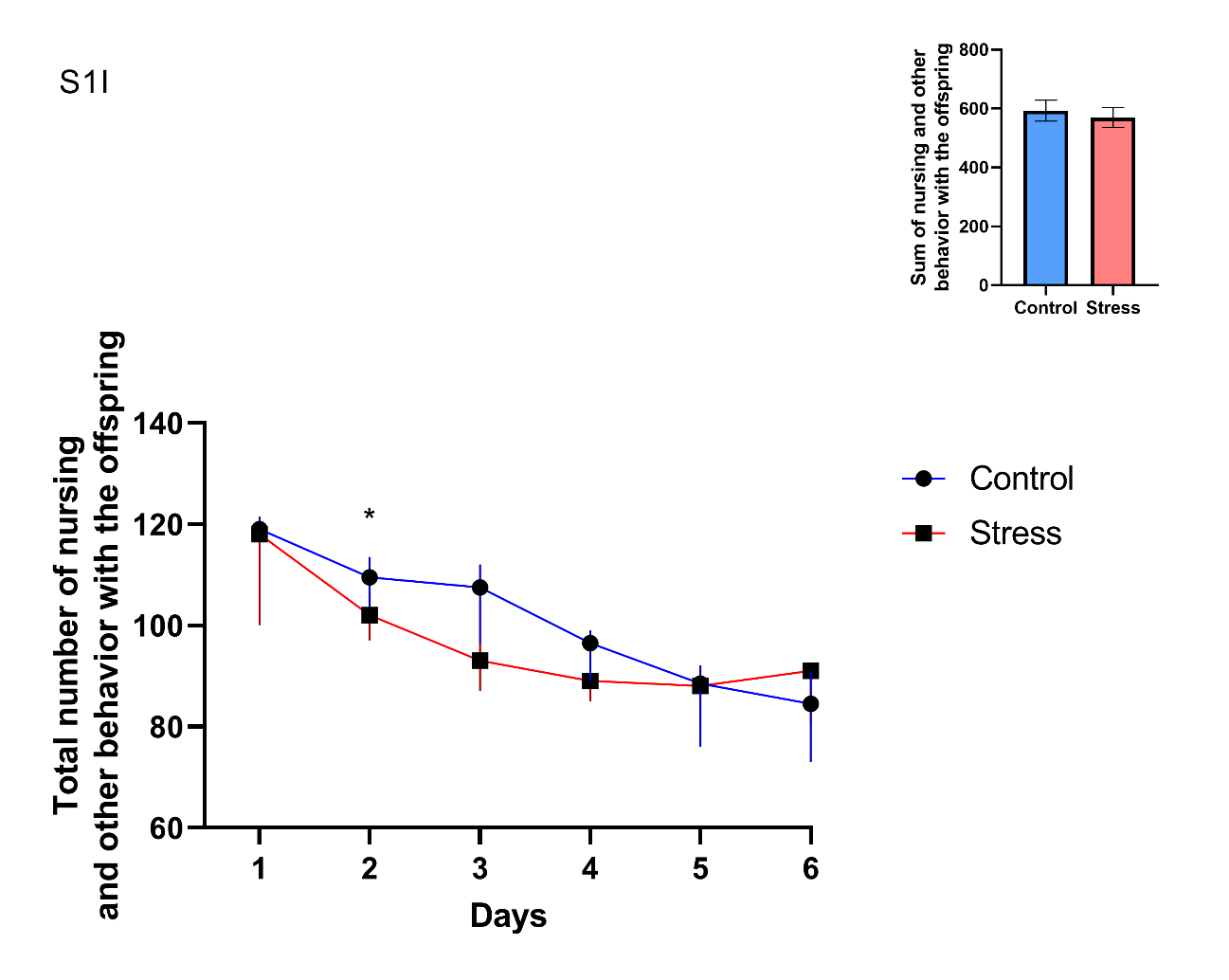
**

**
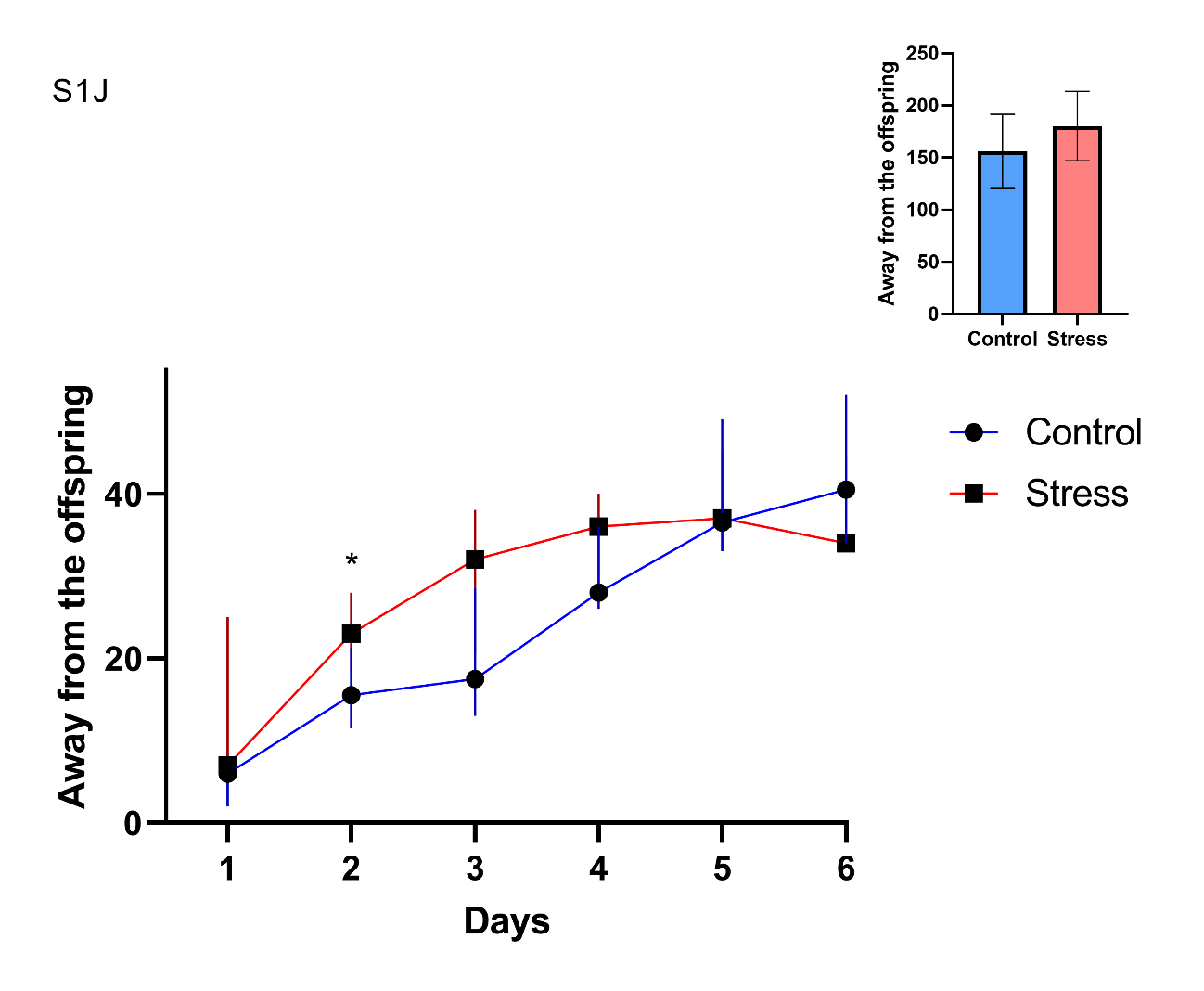
**

**Supplementary Figure 1:** Number of maternal care behaviors during 6 postnatal days (PND) (points and lines graph) and the median and (min. and max.) of the sum of six days' behaviors (bar graphs). The maternal care was evaluated through the analysis of the number of passive nursing (**S1A**), blanket nursing (**S1B**), arched-back nursing (ABN)1 (**S1C**), ABN2 (**S1D**), ABN3 (**S1E**), non-nursing contact (**S1F**), cleaning the offspring (**2G**), carrying the offspring to the nest (**S1H**), the total number of nursing and other behaviors (**S1I**), and away from the offspring (**S1J**). Data are represented as mean ± standard deviation and median with min. to max. values for parametric and non-parametric variables, respectively. *p<0.05 compared to the control group, according to Student's T-test or Mann-Whitney U test.

**Supplementary Figure 2** – IL-6 Offspring

No changes were seen in the levels of IL-6 in the frontal cortex, hippocampus, striatum, and serum in all the experimental groups.

IL-6 Frontal Cortex sex F (1, 32) = 0.09307, p=0.7623 prenatal stress F (1, 32) = 8.786, p=0.0057 treatment F (1, 32) = 0.6379, p=0.4304 sex vs. prenatal stress F (1, 32) = 1.883, p=0.1796 sex vs. treatment F (1, 32) = 0.1271, p=0.7238 prenatal stress vs. treatment F (1, 32) = 0.07658, p=0.7838 interaction F (1, 32) = 0.02736, p=0.8697; Hippocampus H=13.93 p=0.0525; Striatum sex F (1, 32) = 42.43, p<0.0001 prenatal stress F (1, 32) = 4.824, p=0.0354 treatment F (1, 32) = 0.01391, p=0.9068 sex vs. prenatal stress F (1, 32) = 0.08416, p=0.7736 sex vs. treatment F (1, 32) = 5.170, p=0.0298 prenatal stress vs. treatment F (1, 32) = 3.984, p=0.0545 interaction F (1, 32) = 1.867e-006, p=0.9989. Serum sex F (1, 32) = 1.336, p=0.2562 prenatal stress F (1, 32) = 2.968, p=0.0946 treatment F (1, 32) = 0.03384, p=0.8552 sex vs. prenatal stress F (1, 32) = 1.013, p=0.3218 sex vs. treatment F (1, 32) = 0.1749, p=0.6786 prenatal stress vs. treatment F (1, 32) = 1.292, p=0.2642 interaction F (1, 32) = 0.09704, p=0.7574


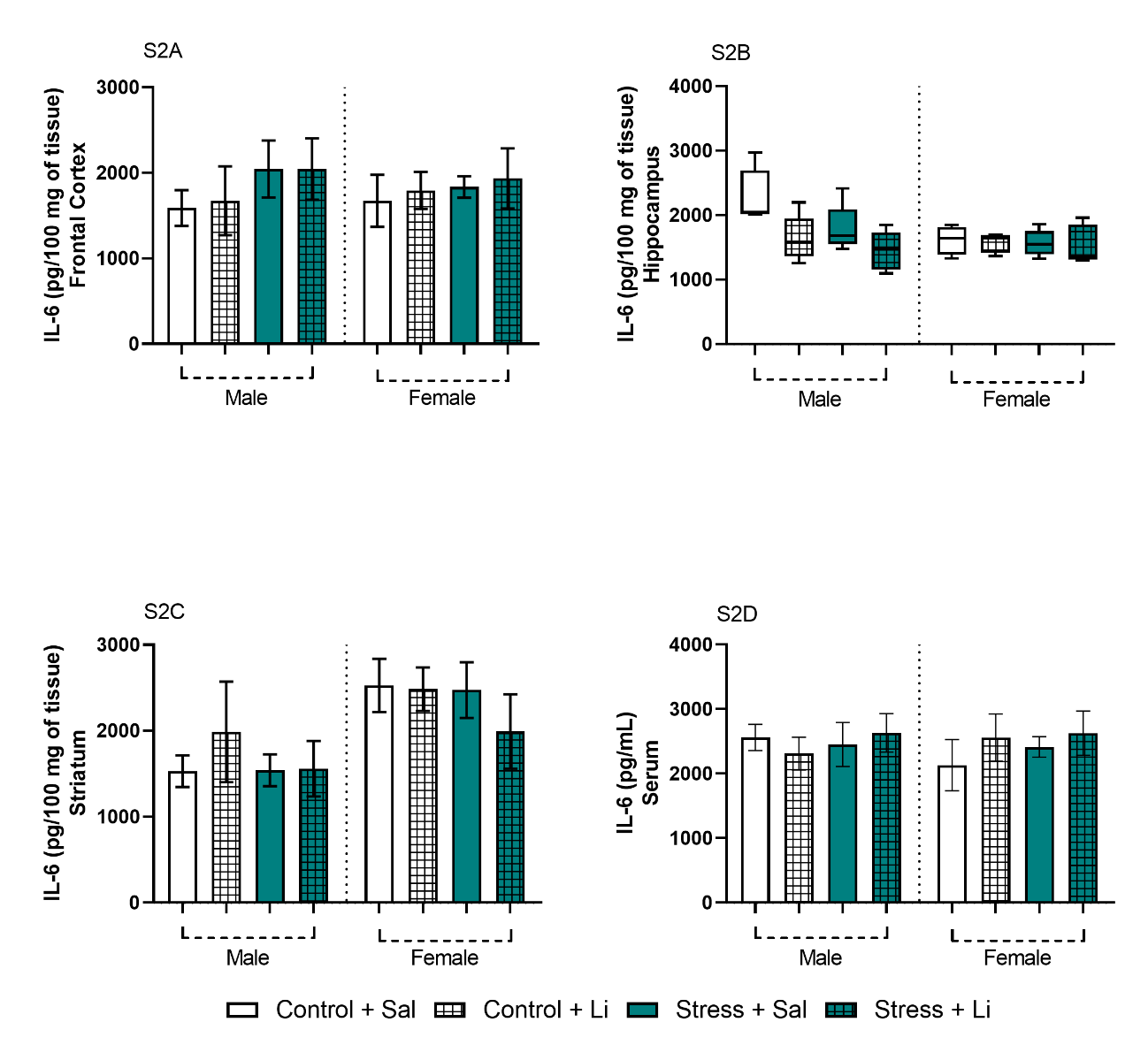


**Supplementary Figure 2:** Levels of interleukin (IL)-6 in the frontal cortex (**S2A**), hippocampus (**S2B**), striatum (**S2C**), and serum (**S2D**) of offspring submitted to prenatal stress and treated with lithium (Li) or saline (Sal). Data are represented as mean ± standard deviation or median with min. to max. values for parametric and non-parametric variables, respectively. *p<0.05 compared to the control + Sal group, # p<0.05 compared to the stress + Sal group, according to two-way ANOVA followed by Tukey’s post hoc or Kruskal-Wallis test followed by the Mann-Whitney U test.

**Supplementary Figure 3** – GPx Offspring

The activity of GPx between groups showed no statistically significant differences. Frontal Cortex H = 19.47 p = 0.0068; Hippocampus H = 10.42 p = 0.1660; Striatum sex F (1, 32) = 9.894, p=0.0036 prenatal stress F (1, 32) = 0.008576, p=0.9268 treatment F (1, 32) = 0.01916, p=0.8908 sex vs. prenatal stress F (1, 32) = 0.0002231, p=0.9882 sex vs. treatment F (1, 32) = 1.528, p=0.2255 prenatal stress vs. treatment F (1, 32) = 2.944, p=0.0959 interaction F (1, 32) = 0.04452, p=0.8342; Serum H = 14.94 p = 0.0368.

**
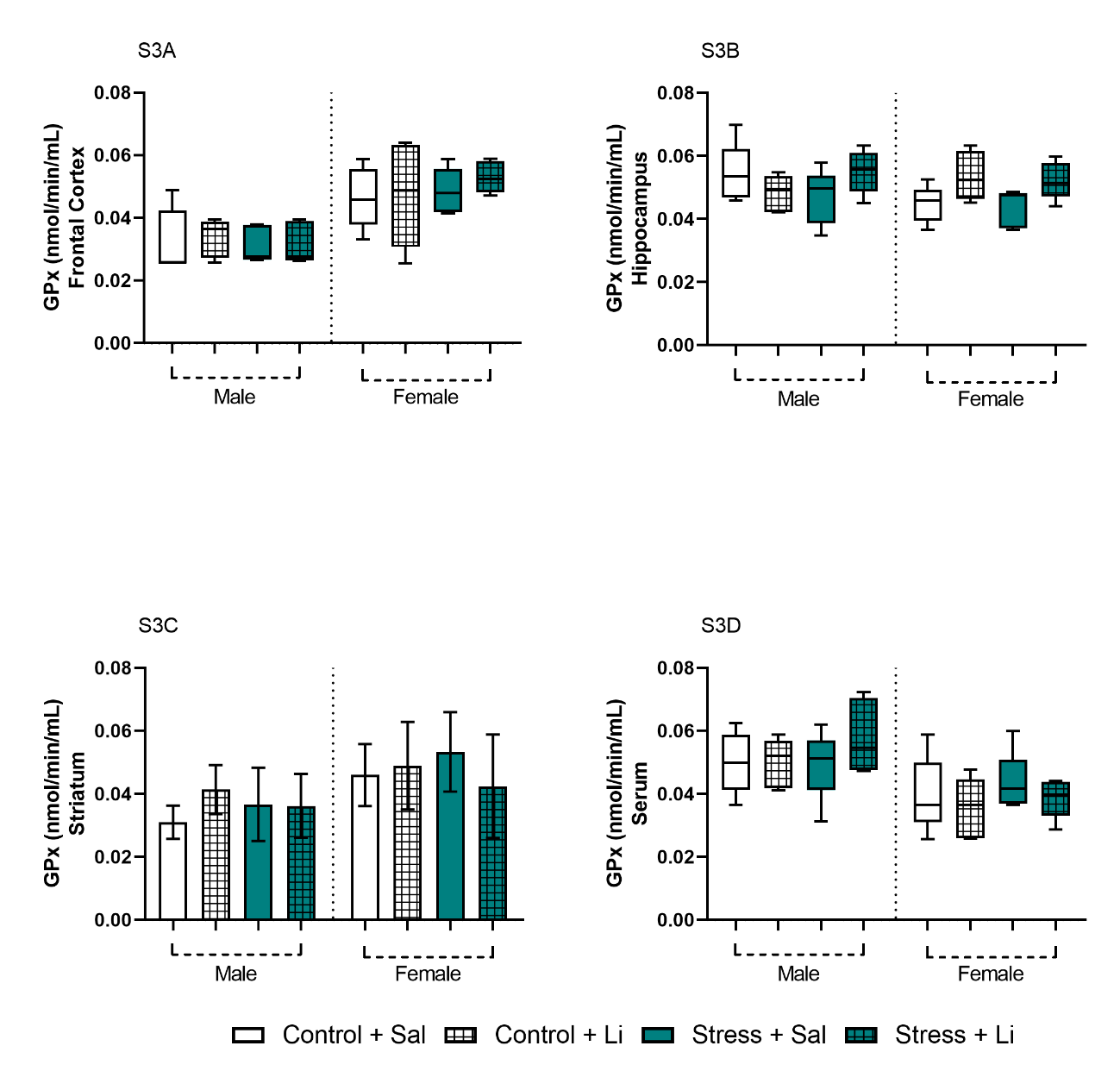
**

**Supplementary Figure 3:**  Activity of glutathione peroxidase (GPx) in the frontal cortex (**S3A**), hippocampus (**S3B**), striatum (**S3C**), and serum (**S3D**) of offspring submitted to prenatal stress and treated with lithium (Li) or saline (Sal). Data are represented as mean ± standard deviation or median with min. to max. values for parametric and non-parametric variables, respectively. *p<0.05 compared to the control + Sal group, # p<0.05 compared to the stress + Sal group, according to two-way ANOVA followed by Tukey’s post hoc or Kruskal-Wallis test followed by the Mann-Whitney U test.

**Supplementary Figure 4** – GR Offspring

The activity of GR between groups showed no statistically significant differences. Frontal Cortex sex F (1, 32) = 0.05718, p=0.8125 prenatal stress F (1, 32) = 1.205, p=0.2805 treatment F (1, 32) = 0.2090, p=0.6506 sex vs. prenatal stress F (1, 32) = 0.1277, p=0.7232 sex vs. treatment F (1, 32) = 0.1032, p=0.7502 prenatal stress vs. treatment F (1, 32) = 1.539, p=0.2238 interaction F (1, 32) = 0.05998, P=0.8081; Hippocampus H =. 7.610 p = 0.3682; Striatum sex F (1, 32) = 0.6924, p=0.4115 prenatal stress F (1, 32) = 0.5128, p=0.4791 treatment F (1, 32) = 0.2746, p=0.6039 sex vs. prenatal stress F (1, 32) = 3.419 p=0.0737 sex vs. treatment F (1, 32) = 0.4080, p=0.5275 prenatal stress vs. treatment F (1, 32) = 0.01890, p=0.8915 interaction F (1, 32) = 0.4200, p=0.5216; Serum sex F (1, 32) = 0.01502, p=0.9032 prenatal stress F (1, 32) = 0.9048, p=0.3486 treatment F (1, 32) = 0.2465, p=0.6229 sex vs. prenatal stress F (1, 32) = 0.002164, P=0.9632 sex vs. treatment F (1, 32) = 0.1377, p=0.7130 prenatal stress vs. treatment F (1, 32) = 0.3988, p=0.5322 interaction F (1, 32) = 0.1347, p=0.7160.

**
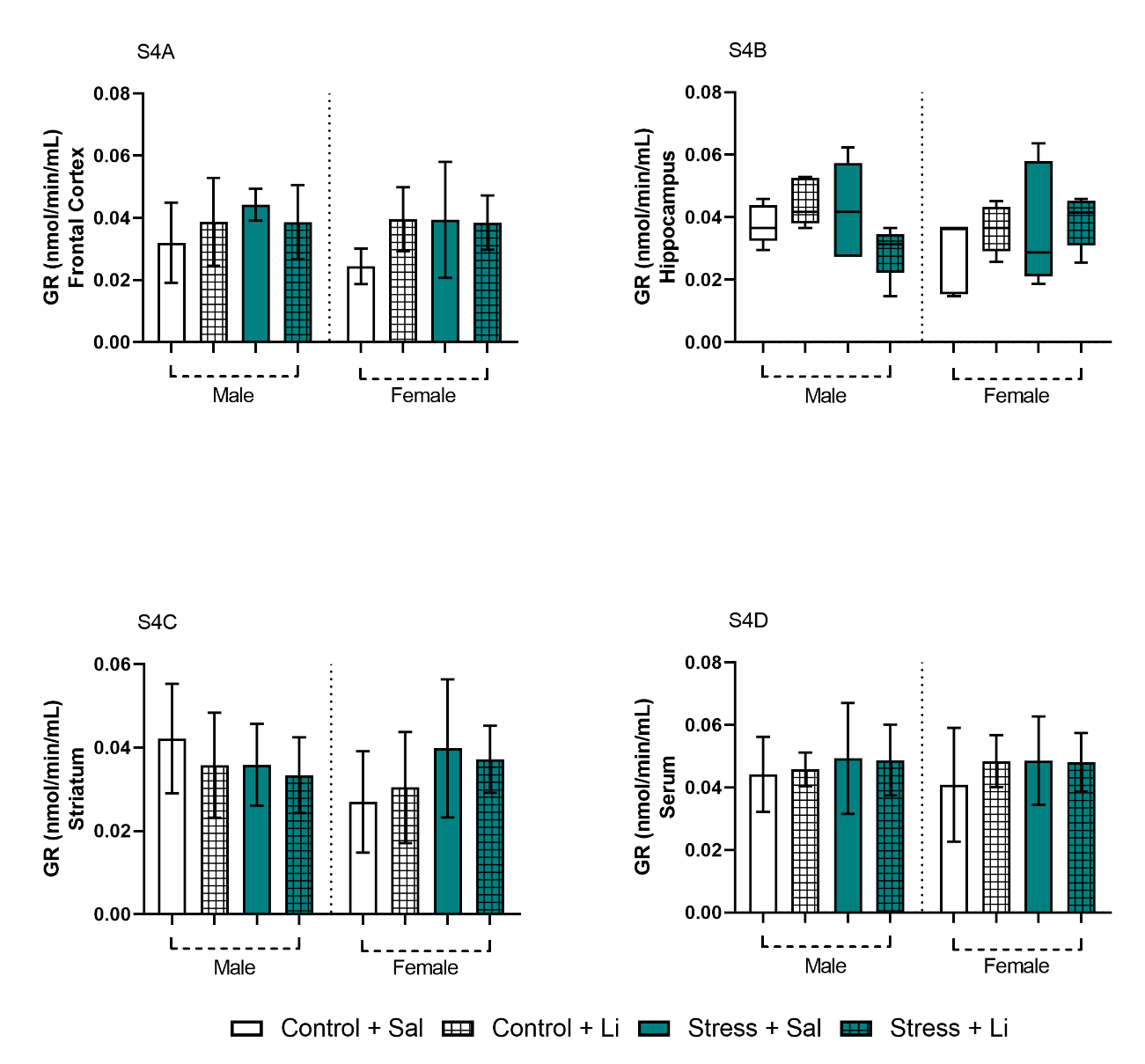
**

**Supplementary Figure 4:** Activity of glutathione reductase (GR) in the frontal cortex (**S4A**), hippocampus (**S4B**), striatum (**S4C**), and serum (**S4D**) of offspring submitted to prenatal stress and treated with lithium (Li) or saline (Sal). Data are represented as mean ± standard deviation or median with min. to max. values for parametric and non-parametric variables, respectively. *p<0.05 compared to the control + Sal group, # p<0.05 compared to the stress + Sal group.
